# Oppositely-charged coordination cages form a type I porous ionic liquid with two pore sizes

**DOI:** 10.1039/d5mh02121a

**Published:** 2026-03-06

**Authors:** Simone Adorinni, Hugh P. Ryan, Lillian Ma, Tanya K. Ronson, Sudhakar Gaikwad, Barbara Rossi, Lucia Nasi, Jonathan R. Nitschke, Silvia Marchesan

**Affiliations:** a Yusuf Hamied Department of Chemistry, University of Cambridge CB2 1EW Cambridge UK jrn34@cam.ac.uk; b Area Science Park 34149 Basovizza Italy; c CNR-IMEM Institute Parco Area delle Scienze 37/A 43124 Parma Italy; d Department of Chemical and Pharmaceutical Sciences, University of Trieste 34127 Trieste Italy smarchesan@units.it; e INSTM, University of Trieste 34127 Trieste Italy

## Abstract

Here we introduce a new type of porous liquid that combines two coordination cages in a single salt. This material is prepared through the ion metathesis of two oppositely-charged cages: one charged +20, and the other with a −16 charge. This ionic liquid has higher porosity than previously reported systems, because both of its components are hollow cages. The enhanced porosity holds promise for improving performance in chemical separations or storage, reflecting the guest-binding selectivity of the two cages, and the applications accessible due to its liquid nature.

## Abstract 2

Oppositely charged metal–organic cages form a neat type-I ionic liquid with dual intrinsic cavities.

## Graphical abstract

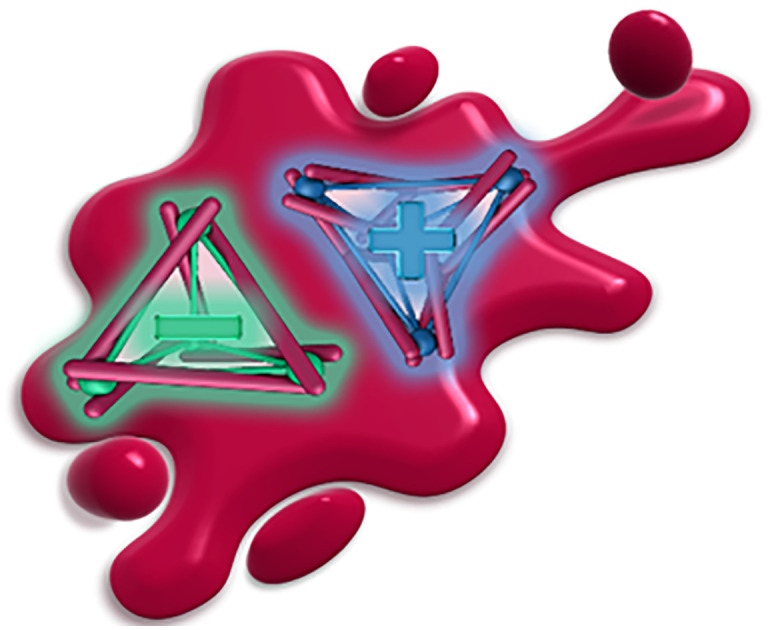

**New concepts**

Porous liquids are an emerging class of materials that combine intrinsic molecular cavities with fluid behaviour. Existing type I porous liquids have relied on a single porous species paired with non-porous counterions, limiting pore density and functional diversity. Here we introduce a new conceptual strategy: pairing two oppositely charged metal–organic cages so that each cage serves as the counterion to the other. This design produces a mixed-cage ionic liquid with two distinct and addressable pore environments, representing a breakthrough in the structural engineering of porous liquids. This dual-porosity system expands free internal volume beyond previous porous ionic liquids and enables selective host–guest binding within each cage type, a capability not accessible through single-cage systems. Because counterion occupation of cavities is eliminated, the accessible pore volume is markedly increased. The liquid nature of this material further provides the potential for guest encapsulation and separation processes to occur without solid–liquid interfaces, while its thermal and rheological stability open opportunities for transport and processing. This concept provides an unprecedented platform for designing multifunctional porous liquids that combine tailored cavity environments with solution-phase mobility, offering new routes to molecular separation, chemical storage, and catalysis.

## 1 Introduction

The engineering of well-defined, intrinsic pores in liquids has enabled the emergence of a novel class of materials recently, combining the virtues of both liquids and solids.^1,2^ These new materials have potential industrial applications that include chemical separations, purifications, and gas storage.^3–5^ Intrinsic porosity refers to rigid molecules that have permanent cavities within them. This type of porosity differs from the extrinsic type, referring to the small and transient cavities that exist between the molecules of any liquid.

**Fig. 1:**
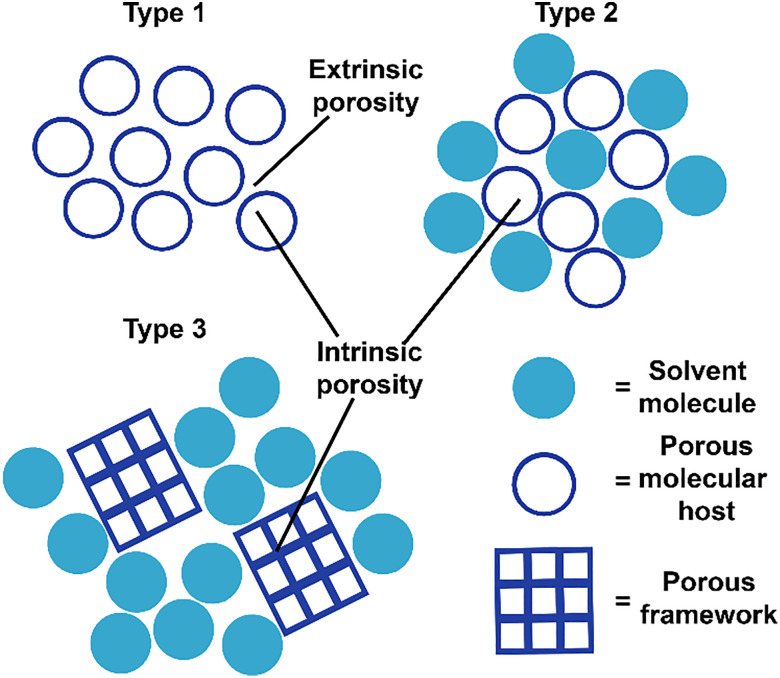
Graphical representation of the three types of porous liquids proposed by James.^6^

The concept of liquid materials with intrinsic porosities was first postulated by James in 2007.^6^ James proposed three types of liquid phases with permanent porosity (Fig. 1).^6^ Type I liquids have intrinsic porosity in the neat state.^1^ Type II liquids consist of a rigid host dissolved in a solvent that does not penetrate its pores, whereas in type III, a solid microporous framework material, such as a zeolite or a metal–organic framework, is suspended in a solvent that does not permeate the pores.^6^

Metal–organic cages have been employed as porous solid materials for applications that exploit their hollow cavities.^7–10^ A few such cages have recently been utilized in the synthesis of type I and type II porous liquids. The first examples of type II porous liquids involved the use of an imine-based organic cage with the crown ether 15-crown-5 as a sterically-hindered solvent.^11^ Recently, a novel example of a porous liquid able to transform from type III to type II was reported, based upon the disassembly of framework materials consisting of Rh^II^-based cuboctahedral cage subunits.^12^

In contrast, the synthesis of type I porous liquids remains challenging, due to the requirement that the porous component be a liquid near room temperature. This class of porous liquids is nonetheless attractive, due to their lower intrinsic volatility and potentially higher pore volumes.^1^ Initial strategies involved the alkylation of a porous organic imine-based cage, as increasing the alkyl chain length decreased the melting point. Another design strategy involved anionic cages and a sterically-hindered cationic component.^13,14^ We reported an ionic liquid where the cationic component was permanently porous, consisting of a Zn^II^_4_L_4_ tetrahedral cage appended with poly(ethylene glycol) (PEG)-imidazolium chains, which rendered it liquid in its neat state with a free volume per gram of 3.94 mm^3^.^15^ This type I porous liquid was capable of binding a diverse range of guests, which could be recovered under reduced pressure.

## 2 Results and discussion

### 2.1 Synthesis and structural characterisation of the coordination cages

**Fig. 2:**
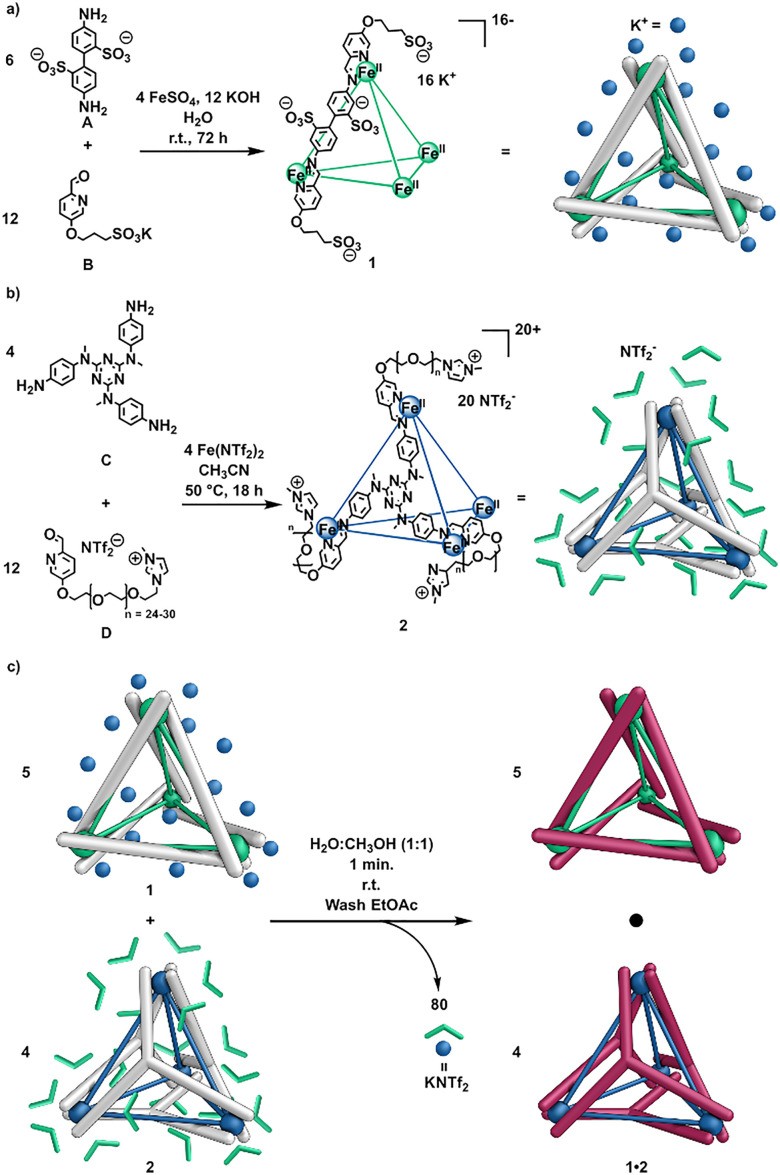
(a) Subcomponent self-assembly of the potassium salt of anionic cage 1. (b) Subcomponent self-assembly of cationic cage 2 as the triflimide salt. (c) Ion metathesis between 1 and 2 yielded hetero-cage 1·2 and potassium triflimide.

We hypothesized that a negatively-charged coordination cage would be able to act as the counterion for a positively-charged liquid cage, thereby forming an ionic liquid with greater overall porosity due to its integration of two porous components. Employing two cages as counterions for each other also sidesteps the problem of counterions binding within cages and competing with guests, thus enhancing the free volume available for guest binding. The chosen anionic component was based on a previously reported cage that incorporated anionic central subcomponent A (Fig. 2a).^16^ The negative charge of the parent cage was further increased by the introduction of sulfonated formylpyridine B,^17^ resulting in the formation of Fe^II^_4_L_6_ tetrahedral cage 1 with an overall charge of −16 (Fig. 2a).

**Fig. 3:**
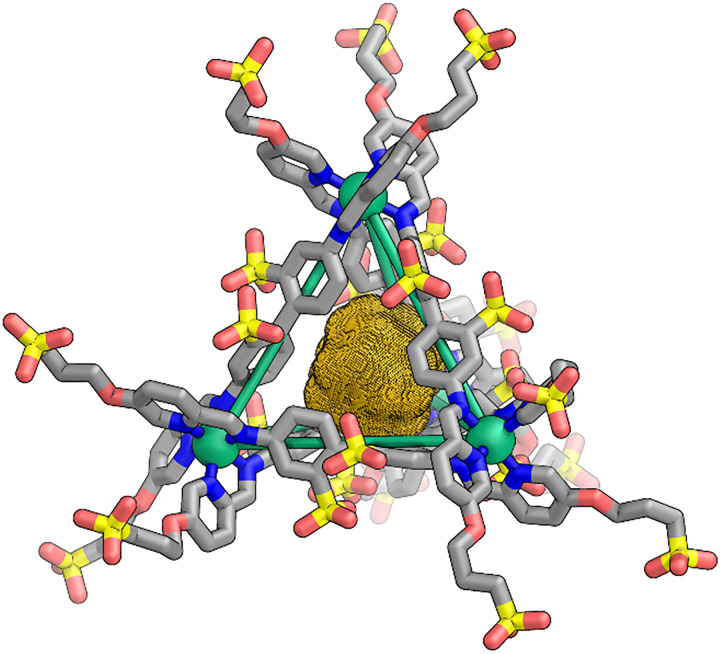
Single-crystal X-ray structure of cage 1 (grey – C, blue – N, red – O, yellow – S, green – Fe), with the MoloVol-calculated void space within the cavity shown as a dark yellow mesh.^23^ Disorder, counterions, hydrogens, and solvent molecules are omitted for clarity.

Single crystals of 1 suitable for X-ray diffraction (Fig. 3) were obtained by vapor diffusion of acetone into an aqueous solution, confirming the formation of the polysulfonated cage framework.^18^ Cationic cage 2 (Fig. 2b) is a Fe^II^_4_L_4_ tetrahedron incorporating triazine-based central subcomponent C and PEG-imidazolium substituted formylpyridine D (*n* = 24–30) peripherally.^19^ Its iron(ii) vertices are less labile than the zinc(ii) vertices employed in a prior ionic liquid cage,^15^ presenting an advantage in terms of stability. In addition, the larger central triazine panel increases the diversity of prospective guest species.^19^ Following an approach similar to that employed in Bloch's pioneering preparation of a two-cage salt,^20,21^ ion metathesis (Fig. 2c) was performed between cages 1 and 2. Five equivalents of 1 in water were combined with 4 equivalents of 2 in methanol for charge balance, and the resulting mixture was washed with ethyl acetate to remove the potassium triflimide byproduct, yielding hetero-cage 1_5_2_4_, hereafter referred to as 1·2 (Fig. 2c).^20,21^ This system constitutes a type I porous liquid, since the cages act as counterions for each other, and they cannot be separated.^6^

The ^1^H NMR spectrum of 1·2 in D_2_O (Fig. S15) included all of the peaks observed separately for cages 1 and 2 in D_2_O. The ^19^F NMR spectrum of 1·2 (Fig. S16b) indicated that the triflimide counterions of cage 2 were removed by washing with ethyl acetate, in contrast to the ^19^F NMR spectrum of 2 in D_2_O (Fig. S16a). EDS mapping also confirmed the successful removal of the NTf_2_^−^ and the K^+^ counterion (Fig. S19), which by contrast could be detected in samples of the parent cages (Figs. S20 and S21).

The NMR spectra of 1·2 in D_2_O thus confirmed its composition as a two-cage salt. Considering only the central cage cavities, the free void volume of 1 in the crystal is 13.6 mm^3^ g^−1^, while that of a previously reported cage, assembled from the same central triazine subcomponent C as 2, is 5.63 mm^3^ g.^22^ These values, together with the 1_5_2_4_ stoichiometry of the neat cage salt, enable the quantification of void volume per mass of 7.41 mm^3^ g^−1^. This value is nearly double that of the previously-reported ionic liquid cage with a nonporous counterion (3.94 mm^3^ g^−1^).^15^

### 2.2 Porosity and guest binding

The presence of two distinct cavities within 1·2 enables selective guest binding within each. The binding of two guests, CClF_3_ and hexafluorobenzene, was studied both for the two cages individually and for the mixed salt. The ^19^F NMR spectra of 1 and 2 in D_2_O indicated that 1 bound CClF_3_ (Fig. S22) and 2 bound hexafluorobenzene (Fig. S24), but not *vice versa* (Fig. S23 and S26). Subsequent ^19^F NMR analysis of 1·2 demonstrated its capacity for selective encapsulation of these two guests within their corresponding cages, as evidenced by the observation of two signals, at −26.3 ppm (CClF_3_ ⊂1) and −162.3 ppm (hexafluorobenzene⊂2) (Fig. S27). Notably, only 38% of the hexafluorobenzene present bound within 2 (Figs. S24 and S25), due to competition between C_6_F_6_ and the triflimide counterions, which occupy the remaining cage cavities. In contrast, 78% of the hexafluorobenzene added was observed to bind within 1·2 (Figs. S27 and S28), which contained only traces of triflimide. This observation highlights a key advantage of this new class of mixed-cage ionic liquids: because the cages are counterions for each other, no counterions compete for binding within the cage cavities, thus further enhancing the useful free pore volume of the material.

Lusby and co-workers introduced the use of Raman spectroscopy to study host–guest binding and demonstrated that the solid-state results agreed with those obtained in solution.^24^ As the high viscosity of neat 1·2 precluded the use of NMR for guest binding studies, we therefore evaluated guest encapsulation in the neat material by Raman spectroscopy.

The Raman spectrum of the CClF_3_ + hexafluorobenzene⊂1·2 complex exhibited a red shift of 2 cm^−1^ in the C=N and N–Fe stretching bands relative to the unbound cage (Fig. S29), consistent with the guest encapsulation results reported by Lusby.^24^ A fitting analysis of the band near 1600 cm^−1^ showed two sub-components whose frequencies remained constant; however, the relative intensity of the two components varied upon guest binding, consistent with interaction between the guest molecules and the cage.

### 2.3 Thermal behaviour and rheological properties

**Fig. 4:**
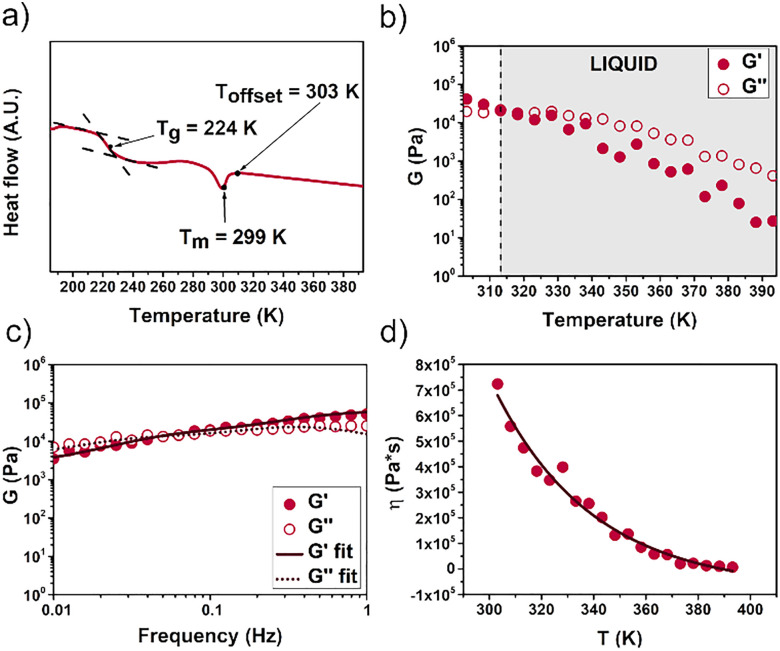
(a) DSC profile (exo up of the 2nd cycle, as the 1st cycle differed from the following three due to the release of water entrapped in the PEG chains, whereas the 3rd cycle recapitulated the 2nd after water removal) of 1·2, with *T*_g_ = 224 K and *T*_m_ = 299 K observed. (b) Temperature-sweep rheological analysis of 1·2 confirmed a liquid phase at temperatures ≥313 K (grey area). (c) Frequency-sweep rheological analysis of 1·2 showed a typical trend for a viscoelastic liquid.^29^ (d) VTF plot for hetero-cage 1·2 (*r*^2^ > 0.98), typical of an IL containing larger cations.^30^

The thermal properties of 1, 2, and 1·2 were probed through TGA and DSC analyses. TGA showed all three materials to have good thermal stability, with decomposition occurring at 573 K (Figs. S30–S32). The DSC trace of 1 (Fig. S33) showed no features beyond an exotherm at 453 K, assigned to cage melting. In contrast, DSC analyses of 2 and 1·2 (Fig. 4a, S34–S36) revealed characteristics associated with PEG derivatives.^25^ The first heating cycle showed features that were not observed in subsequent cycles. These were attributed to the loss of traces of ambient water bound to the PEG chains, which is an established feature of PEG-containing systems.^25^ The glass transition (*T*_g_) and melting (*T*_m_) temperatures were determined from subsequent heating cycles after residual water removal (Figs. S34 and S36). Samples of 2 and 1·2 exhibited glass transition temperatures of 222 K and 224 K, respectively (Fig. 4a, S35). We attribute the absence of a melting peak in the DSC trace of cage 2 to the mobility of the PEG chains (*n* = 24–30).^26^ The nature of the anion is inferred to influence the physical properties of ILs.^27^ In the case of cage 1, its highly symmetric structure and the intermolecular interactions between the cationic and anionic cages gave rise to a *T*_m_ of 299 K for 1·2 (Fig. 4a).^28^

The rheological properties of 1·2 were first probed through time sweep tests at different temperatures. We started from 303 K, corresponding to the offset temperature (*T*_offset_) of the enthalpic peak in the DSC analysis, so as to have stable samples. At temperatures ≥313 K the material was a liquid (Fig. 4b). The higher melting temperature obtained by rheometry was attributed to the lower accuracy of this instrument for assessment of melting temperatures relative to DSC, as observed previously.^31^ The elastic (*G*′) and viscous (*G*″) moduli were observed to decrease as the temperature increased, in accordance with other reported ILs.^32^ The viscoelastic liquid nature of 1·2 was also confirmed by frequency-sweep analysis (Fig. 4c). Our rheological testing yielded a plot typical of a material with Maxwell-type behavior, where at higher frequencies, *G*′ is larger than *G*″. On the contrary, at lower frequencies, the situation reversed, reflecting the liquid nature of the material.^29^

One of the key parameters for applications of ILs is the temperature-dependence of their viscosity.^28^ Generally, the viscosity (*η*) of ILs containing PEG moieties decreases as the temperature rises, and can be modeled using a simple linear Arrhenius dependence.^30^ However, 1·2 fitted poorly to this model (Fig. S37), displaying instead a noticeable curvature in temperature–viscosity plots. The downward curvature observed was typical of ILs that fit the Vogel–Tamman–Fulcher (VTF) model (Fig. 4d).^30^ ILs that exhibit similar behavior contain cations with large sizes and short alkyl groups (*e.g.* methyl) attached to positively-charged atoms, as with cage 1, the cationic component of the 1·2 IL.^30^ Shear-rate rheometry revealed Newtonian fluid behavior for 1·2 between 0.001 and 0.1 s^−1^, above which the viscosity decreased (Fig. S38). The observed decrease in viscosity beyond 0.1 s^−1^ indicated the presence of non-Newtonian shear thinning behavior, as observed in other ILs.^33^

### 2.4 Spectroscopic analysis and stability

**Fig. 5:**
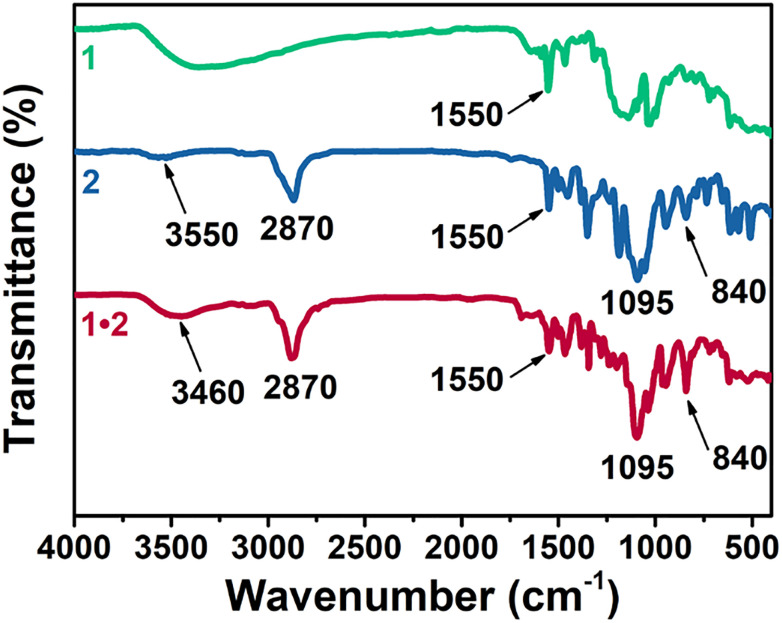
Attenuated total reflectance IR spectra of anionic cage 1, cationic cage 2, and mixed cage salt 1·2. Assignments and an expansion of the region between 3300 and 3000 cm^−1^ is provided in Figs. S39 and S40.

Infrared (IR) spectra of all cages (Fig. 5) showed distinctive bands at 1550 cm^−1^ attributed to C=N stretching, red-shifted from the typical wavenumber of the imine bond (1640–1690 cm^−1^) due to iron coordination.^34,35^ In the region between 700–800 cm^−1^ of cage 2, there are intense bands assigned to the IR-active vibrational modes of the Tf_2_N^−^ counterion.^36^ The absence of these bands in 1·2 further confirmed successful salt metathesis. Furthermore, 1·2 displayed similar modes to cationic cage 2, as shown in Fig. S39. The signals observed in the region between 3000 and 3300 cm^−1^ (Fig. S40) were attributed to the stretching vibrations of the methyl imidazolium cation (mim^+^) moieties and were found to be influenced by its interaction with counterions.^37^ Cage 2, which has Tf_2_N^−^ counterions, displayed a spectral signature in this region similar to that reported for imidazolium-based ILs. The hetero-cage 1·2, whose cationic imidazolium groups were paired with the sulfonate anionic groups of cage 1, exhibited a red shift of these bands (Fig. S40), thereby confirming the presence of a different counterion that paired more strongly with mim^+^ than Tf_2_N^−^.^37^

Visible Raman spectra of all cages displayed features in the range 1450–1650 cm^−1^ (Fig. S41). These signals were attributed to C=N and N→Fe stretching vibrations, as well as to the Raman-active aromatic modes of the ligand.^38^ Resonance Raman spectra collected using excitation at 248 nm enhanced the aromatic signals of the cages, observed as a dominant band at 1595 cm^−1^; no significant differences were observed among the three cages (Fig. S42). Analyses of 1·2 performed at 298 K, 303 K, 313 K, 333 K, 353 K, and 393 K (Fig. S45) showed no changes in the bands corresponding to aromatic vibrational modes. This result confirmed the stability and integrity of 1·2 during rheological and thermal analyses, thus allowing us to infer that melting was associated only with the ionic liquid nature of the hetero-cage and not its decomposition. Analogous temperature-independent spectra were obtained for the two precursors, cages 1 and 2 (Figs. S43 and S44).

## 3 Conclusion

In conclusion, our study introduces and realizes the concept of a porous ionic liquid composed solely of two distinct oppositely-charged metal–organic cages. The inclusion of these cages enabled the void volume of the material to be increased beyond what previously reported systems have achieved, where only one cage formed an ionic liquid.^15^ This dual-porosity ionic liquid opens possibilities for applications in different fields. Host–guest studies revealed that the two components can selectively bind two different types of guests in solution, enhancing their appeal for molecular extraction,^39,40^ separation,^40,41^ and catalysis.^28,42–45^ Future work will explore the uses of this new class of materials for these applications.

## Author contributions

Conceptualization (JRN, SM), investigation (SA, HPR, LM, TKR, BR), resources (BR, JRN, SM), visualization (SA, HPR, LM), writing – original draft preparation (SA), writing – review & editing (SA, HPR, LM, TKR, BR, JRN, SM).

## Conflicts of interest

There are no conflicts to declare.

## Supplementary Material

MH-013-D5MH02121A-s001

MH-013-D5MH02121A-s002

## Data Availability

Any additional data related to this paper may be requested from the authors. All data supporting the findings of this study are available within the article and its supplementary information (SI). Supplementary information: detailed experimental procedures; NMR characterizations; mass spectrometry data; additional spectroscopic, rheological, thermal, microscopic, host–guest data. See DOI: https://doi.org/10.1039/d5mh02121a. CCDC 2177314 contains the supplementary crystallographic data for this paper.^18^
