# Supplementary material for: Oppositely-charged coordination cages form a type I porous ionic liquid with two pore sizes
Source: Mater Horiz. 2026 Mar 6;13(10):5004–9. doi: 10.1039/d5mh02121a (PMC13001717; doi:10.1039/d5mh02121a)
Supplement: MH-013-D5MH02121A-s001 [file MH-013-D5MH02121A-s001.pdf]

Supplementary Information (SI) for Materials Horizons.
This journal is © The Royal Society of Chemistry 2026

Electronic Supplementary Information (ESI)

Oppositely-charged coordination cages form a Type I porous ionic liquid with two pore sizes

Simone Adorinni,[‡, a] Hugh P. Ryan,[‡, a] Lillian Ma,[a] Tanya K. Ronson,[a] Sudhakar Gaikwad,[a] Barbara Rossi,[b] Lucia Nasi,[c] Jonathan R. Nitschke[*,a], and Silvia Marchesan*[*,e,f]*

[a]Yusuf Hamied Department of Chemistry, University of Cambridge, CB2 1EW Cambridge, United Kingdom

[b]Area Science Park, 34149 Basovizza, Italy

[c]CNR-IMEM Institute, Parco area delle Scienze 37/A, 43124 Parma, Italy

[d]Dep. of Chemical and Pharmaceutical Sciences, University of Trieste, 34127 Trieste, Italy

[e]INSTM, University of Trieste, 34127 Trieste, Italy

[‡] These authors contributed equally to this work

* Corresponding authors: jrn34@cam.ac.uk (J.R.N.); smarchesan@units.it (S.M.)

Table of Contents

Table of Contents 2
1) Materials and methods 3
1.1) General 3
1.2) Mass Spectrometry (MS) 3
1.3) Nuclear Magnetic Resonance (NMR) 3
1.4) Transmission Electron Microscopy-Energy Dispersive X-ray Spectroscopy (TEM-EDS) 3
1.5) Solution phase host-guest chemistry 4
1.6) Neat phase host-guest chemistry 4
1.7) Thermogravimetric Analysis (TGA) 4
1.8) Differential Scanning Calorimetry (DSC) 4
1.9) Oscillatory rheometry 4
1.10) Attenuated total reflectance infrared spectroscopy (ATR-IR) 5
1.11) Visible Light Raman Spectroscopy 5
1.12) UV-Resonance Raman (UVRR) Spectroscopy 5
2) Synthesis 6
2.1) Subcomponent A, 4,4'-diamino-[1,1'-biphenyl]-2,2'-disulfonic acid 6
2.2) Subcomponent B, Potassium 3-((6-formylpyridin-3-yl)oxy)propane-1-sulphonate 6
2.3) Subcomponent C, N2,N4,N6-tris(4-aminophenyl)-N2,N4,N6-trimethyl-1,3,5-triazine-2,4,6-triamine 6
2.4) Subcomponent D 6
2.5) Cage 1 11
2.6) Cage 2 14
2.7) Hetero-cage 1•2 16
3) Single-crystal X-ray diffraction 18
4) TEM-EDS 21
5) Host-guest chemistry in solution 23
6) Host-guest chemistry in neat state 27
7) Thermogravimetric analysis 27
8) Differential Scanning Calorimetry (DSC) 29
9) Oscillatory rheometry 30

10) Attenuated total reflectance infrared Spectroscopy 31

11) Raman Spectroscopy 33

References: 35

CRediT: 36

1) Materials and methods

1.1) General

All reagents and solvents were obtained from Merck unless otherwise stated.

1.2) Mass Spectrometry (MS)

High resolution electrospray mass spectra (HR ESI-MS) were obtained on a Waters Synapt G2-Si instrument.

1.3) Nuclear Magnetic Resonance (NMR)

^{1}H, ^{13}C, and ^{19}F NMR spectra were recorded on a 400 MHz Avance III HD Smart Probe Spectrometer at 298 K or on a 500 MHz AVIII HD Smart Probe Spectrometer, unless otherwise stated. Chemical shifts (**δ**) are reported in parts per million (ppm). Spectra are referenced to residual solvent peaks unless otherwise stated. Coupling constants (*J*) are recorded in hertz (Hz). The following splitting abbreviations are used: s – singlet, d – doublet, t – triplet, q – quartet, qn – quintet, sx – sextet, m – multiplet, bs – broad singlet.
DOSY spectra were acquired with the pulse program 'ledbpgp2s' from the standard Bruker Topspin 3.2 library on a 400 MHz Avance III HD Smart Probe Spectrometer. 'ledbpgp2s' is a 2D sequence for diffusion measurements using a stimulated echo and longitudinal eddy-current delay (LED) using bipolar gradient pulses for diffusion and 2 spoil gradients. 16 increments using a quadratic gradient ramp (type q) going from 10 to 80% of the gradient systems maximum power of 10 A. Each increment used 16 transients; a 90 degree rotation required a ^{1}H pulse of 400.1324710 MHz applied for 11.1 ms at 13 W. Other pulses defined in the pulse program are derived from these values. A spectral window of 8012.820 Hz was acquired around an irradiation frequency of 400.1324710 MHz, digitising 32768 K points over 2.0447233 seconds. The maximum field strength was 5.01 G/cm A.[1]

1.4) Transmission Electron Microscopy-Energy Dispersive X-ray Spectroscopy (TEM-EDS)

Transmission electron microscopy (TEM) and scanning transmission electron microscopy (STEM) analyses were performed using a JEOL 2200FS microscope operated at 200 kV, equipped with an Oxford Instruments energy-dispersive X-ray spectroscopy (EDS) detector.
All samples were deposited onto holey carbon–coated copper grids by drop-casting dilute solutions. EDS elemental mapping was carried out in STEM mode using a probe size of 1 nm. Acquisition times were kept deliberately short in order to minimize beam-induced damage to the samples
A spurious Si signal was consistently observed in all EDS spectra, originating from instrumental background of the microscope and detector. This contribution does not affect the interpretation of the elemental composition of the samples.

1.5) Solution phase host-guest chemistry

Neat guests (ca. 4 equiv) were added to solutions of **1** and **2**, and **1•2** in D_2O (ca. 1 – 2 mM) and the samples were allowed to equilibrate for ca. 30 minutes at room temperature.

1.6) Neat phase host-guest chemistry

A sample of the neat porous cage was placed in a small vial and exposed to a 50:50 (v/v) solution of $CClF_3$ and hexafluorobenzene in a larger vial via vapor diffusion overnight at 50 °C. Afterwards, the sample was dried under N_2 and analysed by Raman spectroscopy.

1.7) Thermogravimetric Analysis (TGA)

TGA of ca. 1 mg of sample, loaded on a platinum pan, was recorded on a TGA5500 (TA Instruments, Milan, Italy) under nitrogen flow rate of 50 mL, by equilibrating at 100 °C for 20 min, and following a ramp of 10 °C min^{-1} up to 800 °C.

1.8) Differential Scanning Calorimetry (DSC)

Approximately 5 mg of sample was loaded in *Tzero Aluminum Hermetic* pans. DSC were collected with a TA instruments Discovery DSC 250 at a heating rate of 10 °C min^{-1} and a nitrogen flow rate of 50 mL min^{-1}.

1.9) Oscillatory rheometry

Rheological analysis was performed on a Malvern Kinexus Ultra Plus Rheometer (Alfatest Milan, Italy) with a 20 mm stainless steel parallel plate geometry, after sample pre-treatment. The ionic liquid was first freeze-dried to remove water and immediately deposited onto the rheometer plate and analysed with a gap of 0.8 mm. The sample was heated to 120 °C for 30 minutes to remove any residual water. After 30 minutes the sample was cooled to 25 °C and the different analyses were performed.

The temperature sweep analysis was performed through a time sweep recorded for 60 seconds, using a frequency of 0.01 Hz and a controlled stress of 0.1 Pa. The time sweep was recorded every 5 °C from 30 °C to 120 °C, with an equilibration time of 2 minutes. The frequency sweep at 80 °C was recorded from 0.01 to 1 Hz. Viscosity analysis was performed every 5 °C from 30 °C to 120 °C with an applied stress of 0.1 Pa. Shear-dependent rheology was recorded from a strain of 0.1 s^{-1} until the viscosity decreased.

Viscosity analysis was fitted with the Vogel-Tamman-Fulcher (VTF) model using equation (1):

$$\eta = \eta_0 e^{D^* (T_0/(T - T_0)} \tag{1}$$

The parameters η_0, D*, T_0 of the equation were fitted iteratively using the software Curve Expert Professional.[2] The fitting parameter D* is a strength parameter, related to how closely the temperature dependence of viscosity in a supercooled liquid obeys the Arrhenius law.[3] Stronger liquids have a larger D* value, the ILs reported in the literature are between 1-10.[4] η_0 is the limiting high-temperature viscosity, and it is between 1 and 10 Pa • s.[4] Finally, T_0 is the Vogel temperature, and for ILs it is usually between 100 and 200 K.[4]

1.10) Attenuated total reflectance infrared spectroscopy (ATR-IR)

Samples were dried under vacuum overnight after being transferred onto a 1-cm^2 piece of silicon wafer for attenuated total reflectance infrared (ATR-IR) spectroscopy. ATR-IR spectra were recorded at 4 cm^{-1} resolution, 240 scans, with IR Affinity-1S (Shimadzu).

1.11) Visible Light Raman Spectroscopy

Samples were dried under vacuum overnight after being transferred onto a microscope glass slide. Raman analysis was undertaken with an Invia Renishaw microspectrometer (50) equipped with He-Ne laser at 532 nm. The laser was focused using a 50x microscope and the power of the laser was set as required for the sample. At least 10 spectra per sample were acquired to ensure the homogeneity of the samples.

1.12) UV-Resonance Raman (UVRR) Spectroscopy

Samples were dried under vacuum overnight after being transferred onto a microscope glass slide. UVRR measurements have been collected using the integrated PL200 DUV spectrometer (Photon Systems). The excitation wavelength at 248.6 nm was provided by and NeCu transverse excited hollow-cathode gas laser in 40 μs pulses with an average energy of 3.5 μJ. The laser beam was focalized on the sample surface through a 5X deep UV achromatic objective and the scattered light collected in back-scattering geometry. The Raman signal was analysed via a 200 mm focal length Czerny–Turner

spectrometer equipped with a 3600 g/mm holographic grating (dispersion of 2.2 cm^{-1}/pixel) and a 2048 x 128 pixel thermo-electrically cooled CCD array detector. Acetonitrile was used for the calibration of the spectrometer.
The HCP621G precision temperature-controlled hot and cold stage system (INSTEC, Inc.) was used for the temperature-dependent measurements.

2) Synthesis

2.1) Subcomponent A, 4,4'-diamino-[1,1'-biphenyl]-2,2'-disulfonic acid

Subcomponent **A** were supplied by Sigma Aldrich and used without further further purification.

2.2) Subcomponent B, Potassium 3-((6-formylpyridin-3-yl)oxy)propane-1-sulphonate

Potassium 3-((6-formylpyridin-3-yl)oxy)propane-1-sulfonate was synthesised according to a previously reported procedure.[5]

2.3) Subcomponent C, N2,N4,N6-tris(4-aminophenyl)-N2,N4,N6-trimethyl-1,3,5-triazine-2,4,6-triamine

Triazine-based subcomponent **C** was synthesised using a previously reported procedure.[6]

2.4) Subcomponent D

Subcomponent **D** was synthesised according to a modified previously reported procedure.[7]

Subcomponent precursor D1

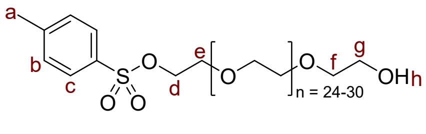

D1

A suspension of polyethylene glycol (M_r ~ 1500 g mol-1, 21.6436 g, 14.4 mmol) in THF (100 mL) was warmed to 35 °C to dissolve the solids. A solution of potassium hydroxide (355.1 mg, 6.329 mmol) in water (3 mL) was added dropwise to the THF solution. p-Toluenesulfonyl chloride (413.9 mg, 2.171 mmol) was then added and the reaction mixture was stirred at 35 °C for 24 hours. The solvent was removed in vacuo and the crude product was extracted with DCM (3 × 10 mL), washed with, HCl (ca. 1.25 M, 5 × 30 mL), water (2 × 30 mL), dried over anhydrous magnesium sulphate, and filtered under gravity and finally the solvent was removed in vacuo. The crude product was purified by flash column chromatography on silica (DCM/MeOH, 7 %) and dried under a stream of nitrogen gas to yield precursor **D1** (1.6347 g, 0.988 mmol, 46 %). ^{1}H NMR (400 MHz, CD_3CN, 298 K, referenced to residual solvent at 1.94 ppm) δ_H = 7.82 (d, J = 8.3 Hz, 2H, H_c), 7.47 (d, J = 8.0 Hz, 2H, H_b), 4.15 – 4.12 (m, 2H, H_d), 3.55 (m, H_g, H_f, H_e, H_{PEG}), 2.79 (s, 1H, H_h), 2.47 (s, 3H, H_a).

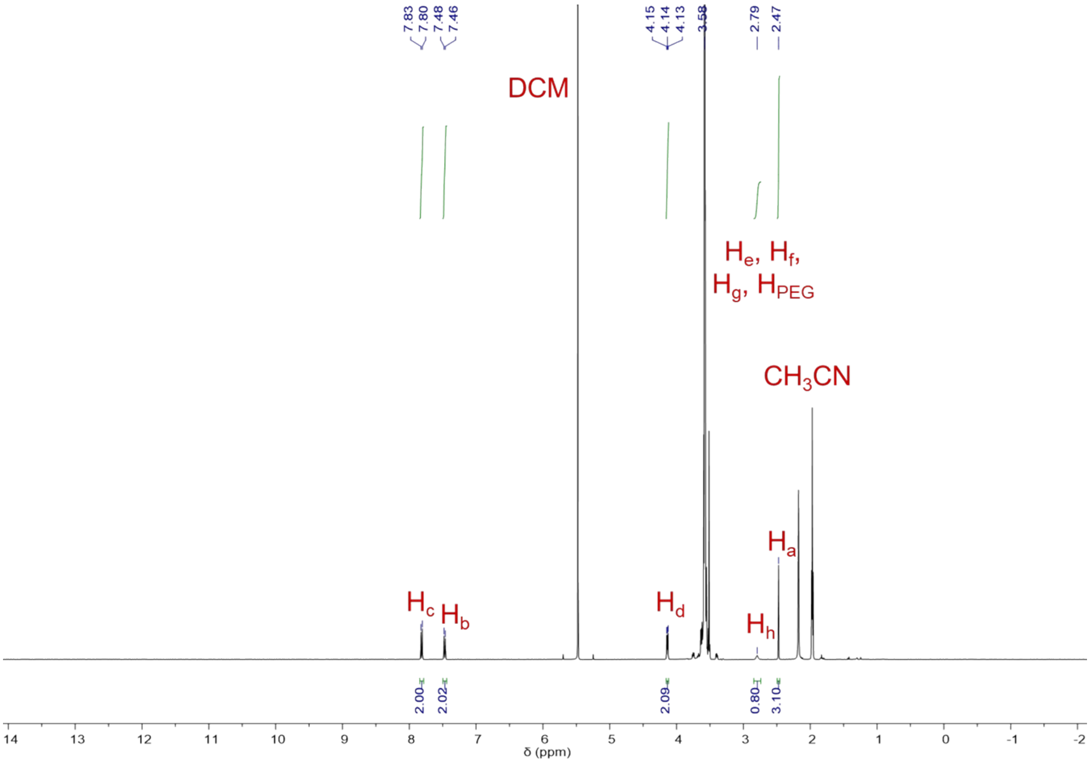

Figure S1: ^{1}H NMR spectrum (400 MHz, 298 K, CD_3CN) of subcomponent **D1**.

Subcomponent precursor D2

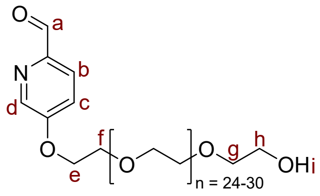

D2

A mixture of precursor **D1** (1.6175 g, 0.978 mmol), potassium carbonate (671.6 mg, 4.859 mmol), sodium iodide (23.8 mg, 0.159 mmol), and 5-hydroxypicolinaldehyde (132.6 mg, 1.077 mmol) in acetonitrile (10 mL) was stirred under nitrogen at 65 °C for 40 hours. The reaction mixture was then filtered under gravity and the solvent removed *in vacuo*. The crude product was extracted with DCM (3 × 10 mL), washed with water (3 × 30 mL), dried over anhydrous magnesium sulphate, and filtered under gravity. Finally, the solvent was removed *in vacuo* and the product dried under a stream of nitrogen gas to yield precursor **D2** (1.3214 g, 0.823 mmol, 84 %). ^{1}H NMR (400 MHz, CD_3CN, 298 K, referenced to residual solvent at 1.94 ppm) δ_H = 9.91 (s, 1H, H_a), 8.45 (d, *J* = 2.8 Hz, 1H, H_d), 7.92 (d, *J* = 8.7 Hz, 1H, H_b), 7.46 (dd, *J* = 8.6, 2.8 Hz, 1H, H_c), 4.30 – 4.26 (m, 2H, H_e), 3.83 (dd, *J* = 5.3, 3.7 Hz, 2H, H_f), 3.55 (m, H_g, H_h, H_{PEG}), 2.78 (t, *J* = 5.7 Hz, 1H, H_i).

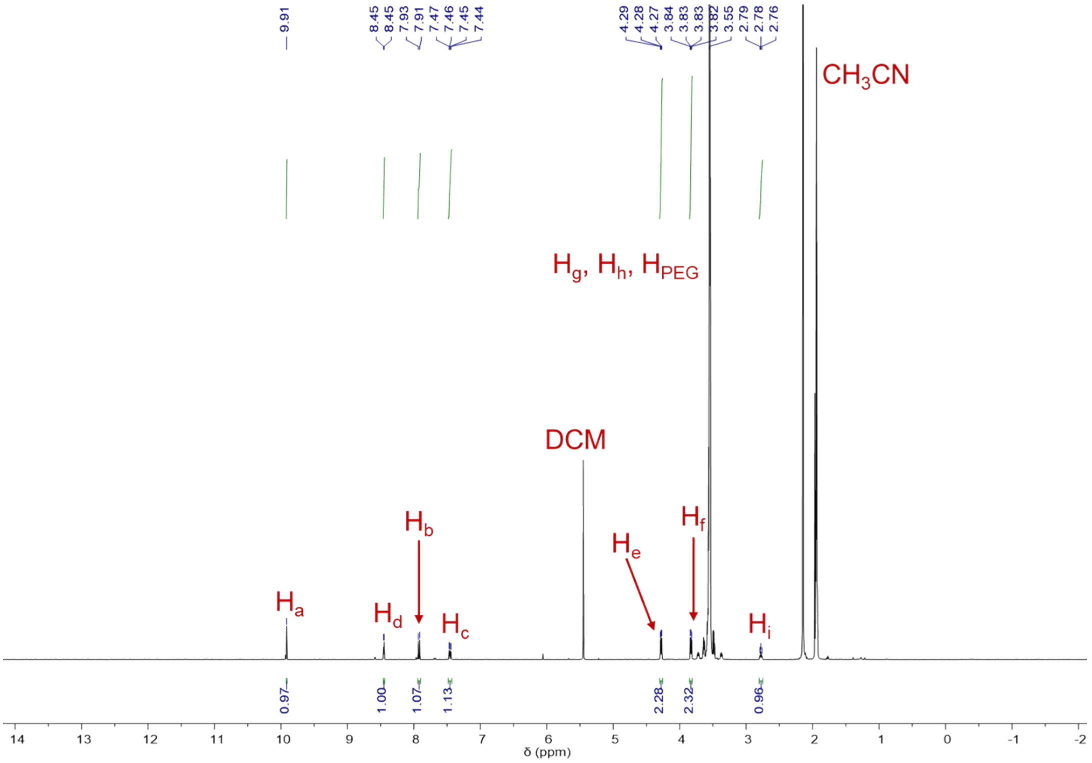

Figure S2: ^{1}H NMR spectrum (400 MHz, 298 K, CD_3CN) of subcomponent **D2**.

Subcomponent precursor D3

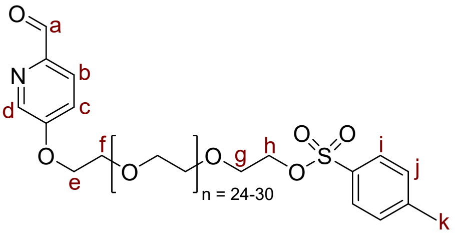

D3

A solution of potassium hydroxide (183.8 mg, 3.276 mmol) in water (1.6 mL) was added dropwise to a solution of **D2** (1.1847 g, 0.738 mmol) in THF (20 mL). p-Toluenesulphonyl chloride (311.8 mg, 1.635 mmol) was then added and the resulting mixture was stirred at room temperature for 22 hours. The solvent was then removed in vacuo and the product was extracted with DCM (3 × 10 mL), washed with water (3 × 30 mL), dried over anhydrous magnesium sulphate, and filtered under gravity. The crude product was purified by flash column chromatography on silica (DCM/MeOH 7 %) and then the product was dried under a stream of nitrogen gas to yield precursor **D3** (1.0325 g, 0.587 mmol, 80 %). ^{1}H NMR (400 MHz, CD_3CN, 298 K, referenced to residual solvent at 1.94 ppm) δ_H 9.91 (s, 1H, H_a), 8.45 (d, *J* = 2.8 Hz, 1H, H_d), 7.92 (d, *J* = 8.7 Hz, 1H, H_b), 7.79 (d, *J* = 8.3 Hz, 2H, H_i), 7.48 – 7.41 (m, 3H, H_c, H_i), 4.30 – 4.26 (m, 2H, H_e), 4.14 – 4.09 (m, 2H, H_f), 3.85 – 3.81 (m, 2H, H_h), 3.55 (m, H_g, H_{PEG}), (2.45 (s, 3H, H_k).

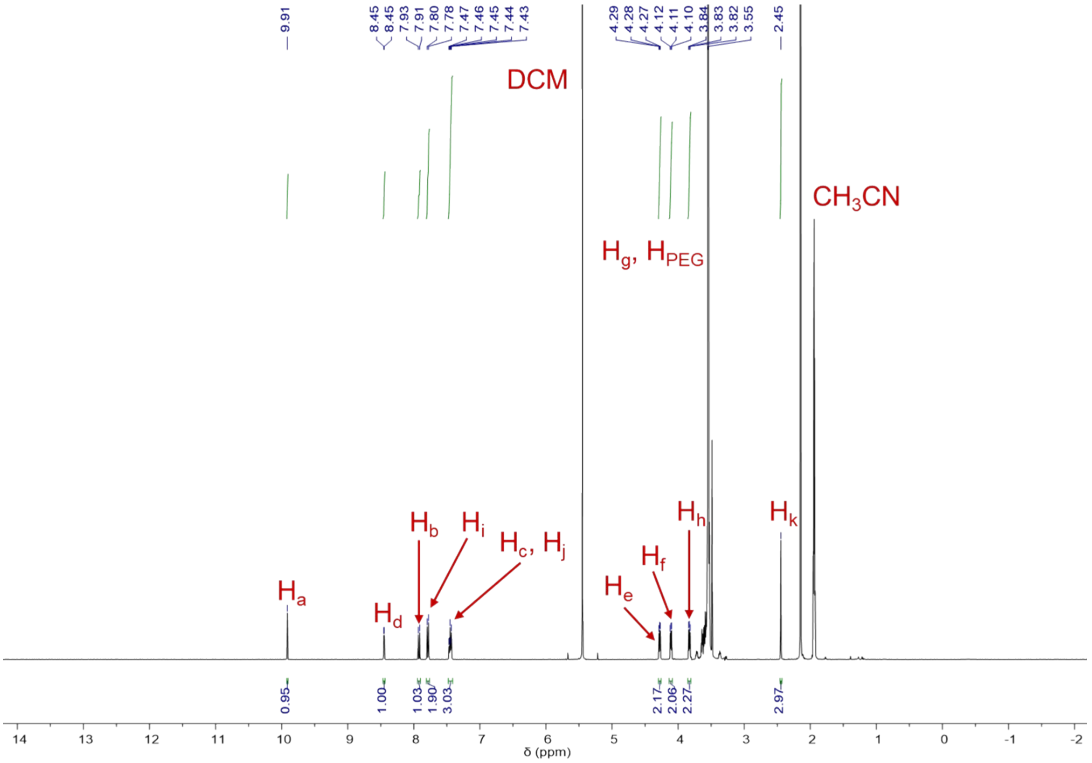

Figure S3: ¹H NMR spectrum (400 MHz, 298 K, CD_3CN) of subcomponent **D3**.

Subcomponent precursor D4

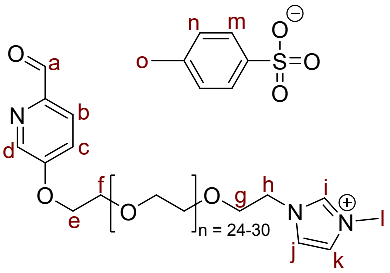

D4

A solution of **D3** (999.3 mg, 0.568 mmol) and 1-methylimidazole (0.25 mL, 3.136 mmol) in acetonitrile (6 mL) was stirred at 65 °C for 40 hours. The solvent was removed *in vacuo* and the crude product was washed with diethyl ether (10 × 30 mL) and dried under a stream of nitrogen gas to yield precursor **D4** (712.3 mg, 0.387 mmol, 68 %). ¹H NMR (400 MHz, CD_3CN, 298 K, referenced to residual solvent at 1.94 ppm) δ_H = 9.92 (s, 1H, H_a), 8.65 (s, 1H, H_i), 8.45 (s, 1H, H_d), 7.92 (d, *J* = 9.1 Hz, 1H, H_b), 7.60 (d, *J* = 7.3 Hz, 2H, H_m), 7.46 (d, *J* = 7.6 Hz, 2H, H_c, H_j), 7.36 (s, 1H, H_k), 7.14 (d, *J* = 7.6 Hz, 2H, H_n), 4.31 – 4.25 (m, 4H, H_e, H_f), 3.85 (s, 3H, H_l), 3.81 – 3.77 (m, 2H, H_h), 3.55 (m, H_g, H_{PEG}), 2.33 (s, 3H, H_o).

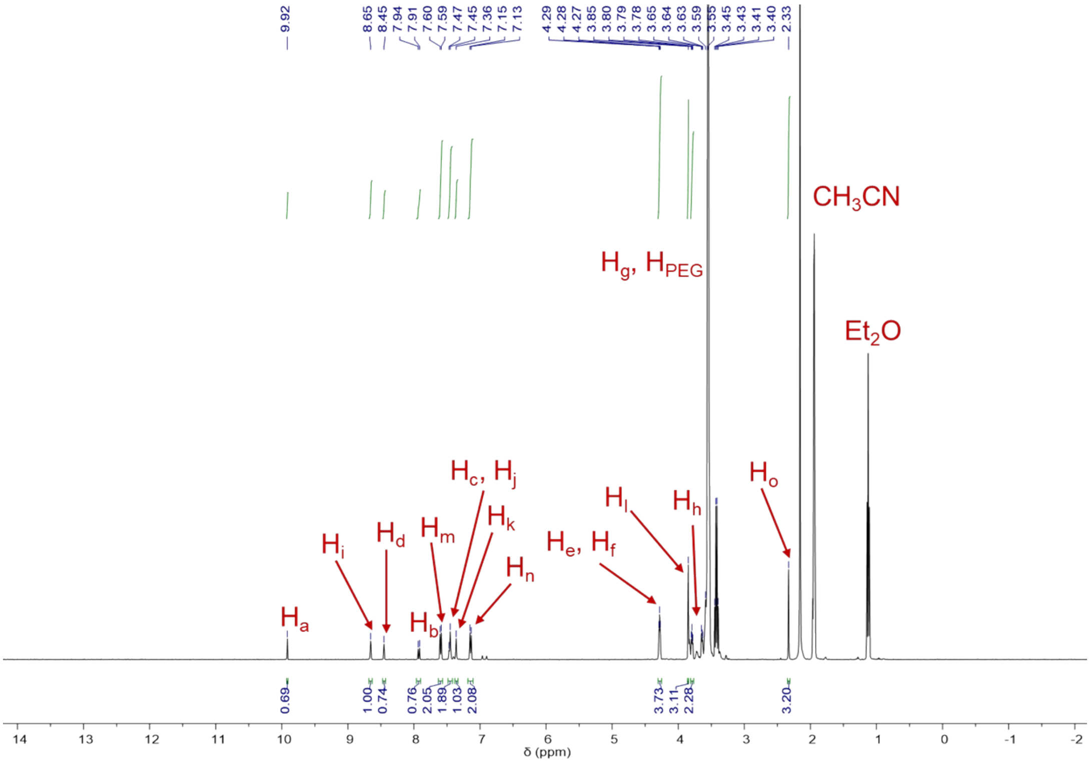

Figure S4: ^{1}H NMR spectrum (400 MHz, 298 K, CD_3CN) of subcomponent **D4**.

Subcomponent D

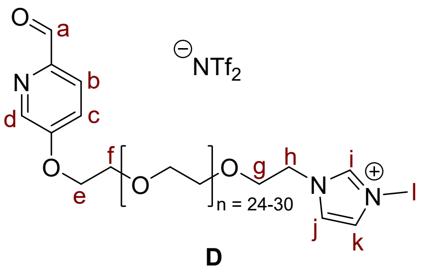

D

A mixture of **D4** (702.8 mg, 0.382 mmol) and lithium triflimide (219.5 mg, 0.765 mmol) in water (5 mL) was stirred at room temperature for 1 minute. DCM (50 mL) was added and the biphasic mixture was then stirred vigorously at room temperature for 1 minute. The phases were then separated and the organic phase was washed with water (3 × 30 mL), dried over anhydrous magnesium sulfate, and filtered under gravity. The solvent was then removed *in vacuo* and the product dried under a stream of nitrogen gas to yield subcomponent **D** (732.5 mg, 0.376 mmol, 98 %). ^{1}H NMR (400 MHz, CD_3CN, 298 K, referenced to residual solvent at 1.94 ppm) δ_H = 9.91 (s, 1H, H_a), 8.60 (s, 1H, H_i), 8.45 (d, J = 2.8 Hz, 1H, H_d), 7.92 (d, J = 8.7 Hz, 1H, H_b), 7.46 (dd, J = 8.9, 2.5 Hz, 2H, H_c, H_j), 7.36 (s, 1H, H_k), 4.28 (td, J = 4.7, 1.9 Hz, 4H, H_e, H_f), 3.85 (s, 3H, H_l), 3.81 – 3.77 (m, 2H, H_h), 3.55 (m, H_g, H_{PEG}).

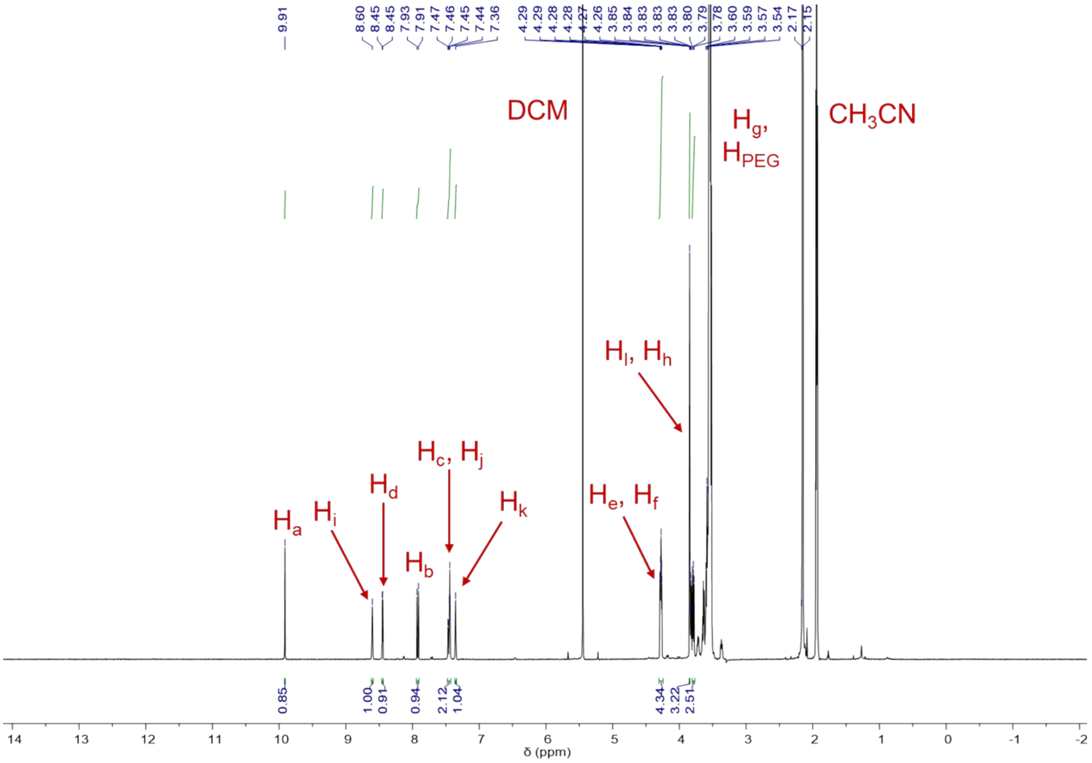

Figure S5: ^{1}H NMR spectrum (400 MHz, 298 K, CD_3CN) of subcomponent **D**.

2.5) Cage 1

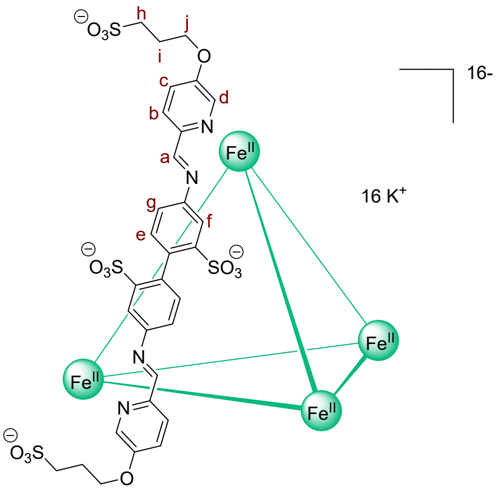

4,4'-diamino-[1,1'-biphenyl]-2,2'-disulfonic acid (39.3 mg, *ca.* 96.3 µmol) (subcomponent **A**), potassium 3-((6-formylpyridin-3-yl)oxy)propane-1-sulfonate (54.4 mg, 192.0 µmol) (subcomponent **B**), potassium hydroxide (12 mg, 213.9 µmol), and iron (II) sulfate heptahydrate (18.4 mg, 66.2 µmol) were dissolved in D_2O (2 mL) and stirred at room temperature for 72 hours. The cage was precipitated with acetone (10 mL), washed with further acetone (10 mL), and dried to air to yield dark pink solid cage **1** (88.6 mg, 15.7 µmol). ^{1}H NMR (400 MHz, D_2O, 298 K, referenced to residual solvent at 4.79 ppm) δ_H = 9.47 (bs, H_a), 8.80 (bs, H_b), 7.97 (bs, H_c), 7.24 (bs, H_d), 7.03 (bs, H_e), 6.44 (bs, H_f), 5.79 (bs, H_g), 4.34 (bs, H_j), 3.07 (bs, H_h), 2.23 (bs, H_i). ^{13}C NMR (125 MHz, D_2O, 298 K) δ_C = 174.0, 159.7, 150.2, 146.0, 142.9, 141.8, 135.7, 133.2, 133.1, 131.9, 131.6, 122.0, 121.8, 121.4, 121.0, 120.0, 117.0, 67.8, 67.2, 47.6, 23.9). HR ESI-MS confirmed the successful formation of **1**, despite the presence of multiple m/z peaks resulting from anion exchange of K^+ with Na^+ and H^+. Crystallography confirms the formation of cage **1** (Figure S13).

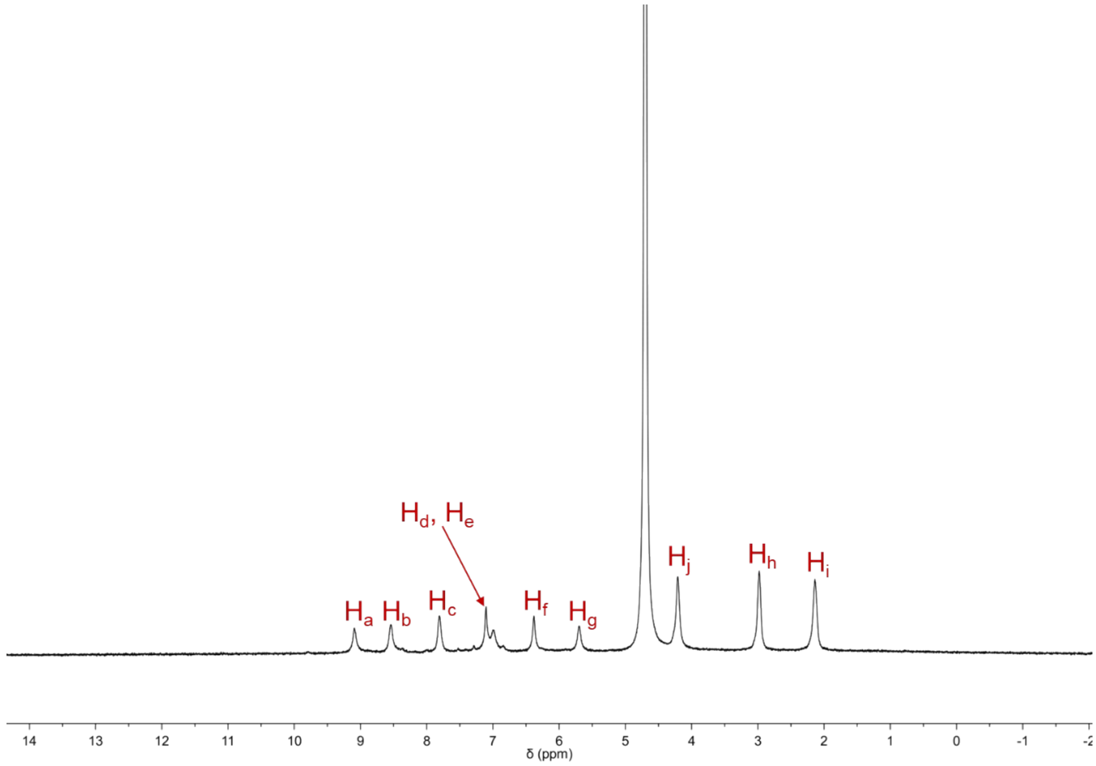

Figure S6: ^{1}H NMR spectrum (400 MHz, 298 K, D_2O) of cage **1**.

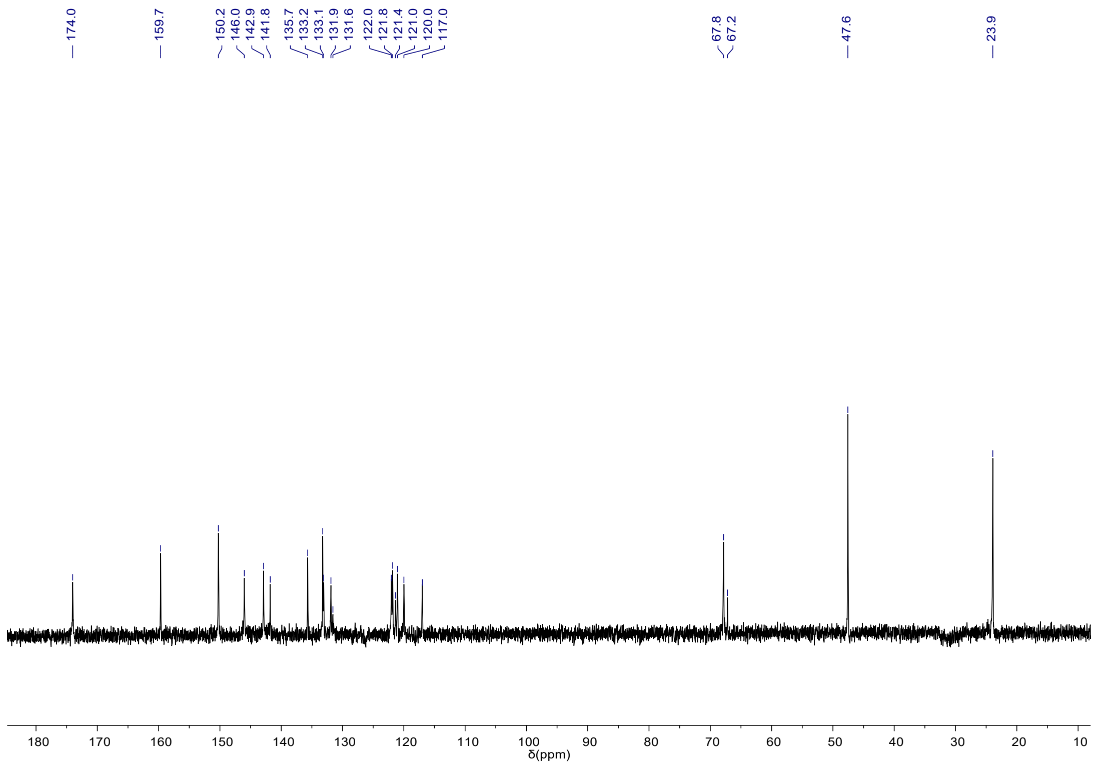

Figure S7: ^{13}C NMR spectrum (125 MHz, 298 K, D_2O) of cage **1**.

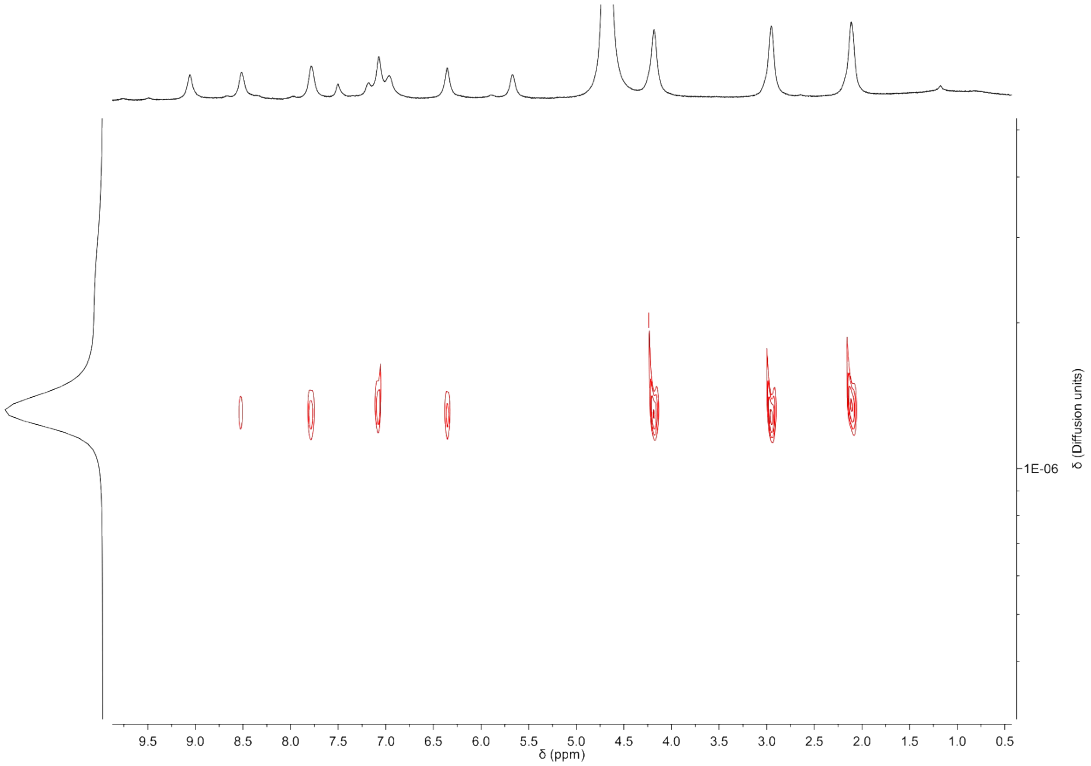

Figure S8: ^{1}H DOSY spectrum of cage **1** in CD_3CN (298 K, 400 MHz), D ~ 1.34×10^{-6} cm^2 s^{-1}.

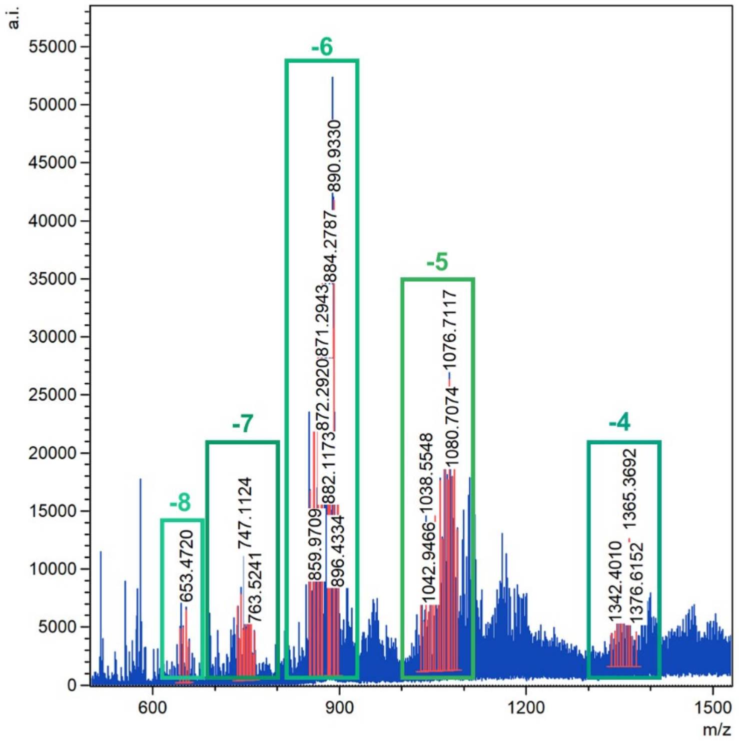

Figure S9: HR ESI-MS of 1. m/z values corresponding to -8, -7, -6, -5, and -4 were observed. The presence of multiple m/z peaks resulting from anion exchange of K+ with Na+ and H+.

2.6) Cage 2

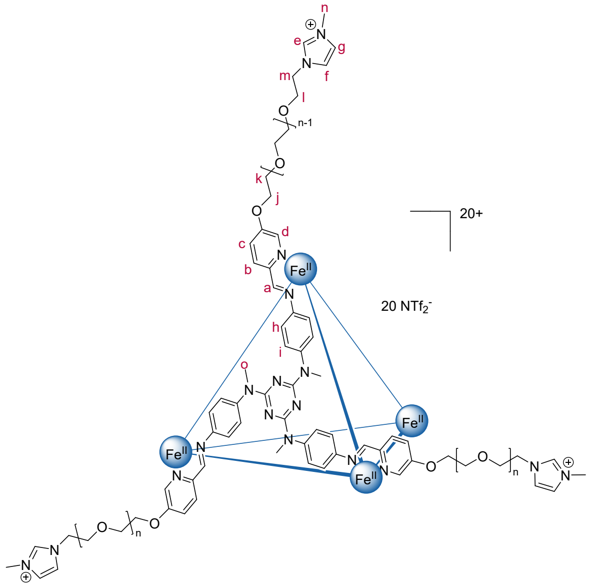

N2,N4,N6-tris(4-aminophenyl)-N2,N4,N6-trimethyl-1,3,5-triazine-2,4,6- triamine (11.7 mg, 26.5 μmol) (subcomponent **C**), subcomponent **D** (115.1 mg, 79.3 μmol), and iron (II) triflimide (18.3 mg, 26.2 μmol) were dissolved in CD_3CN (2 mL) and stirred at 50 °C for 18 hours. The cage was precipitated with diethyl ether (8 mL), washed with further diethyl ether (2 × 5 mL), and dried under a stream of nitrogen gas to yield a dark red sticky liquid cage **2** (114.5 mg, 5.3 μmol, 81 %). ^{1}H NMR (400 MHz, CD_3CN, 298 K, referenced to residual solvent at 1.94 ppm) δ_H = 8.70 (s, H_a), 8.58 (s, H_e), 8.42 (m, H_b), 7.88 (d, 9 Hz, H_c), 7.44 (m, H_f), 7.35 (m, H_g, H_i), 7.01 (s, H_d), 5.08 (m, H_h), 4.27 (m, H_j, H_m), 3.84 (s, H_n), 3.79 (m, H_k, H_l), 3.35 – 3.75 (m, H_o, H_{PEG}).

The broad PEG signals were likely to have been caused by rapid relaxation and this hindered the collection of 2D NMR spectra. The harsh conditions required for mass spectrometry induce decomposition of the cage, thus precluding acquisition of mass spectra.

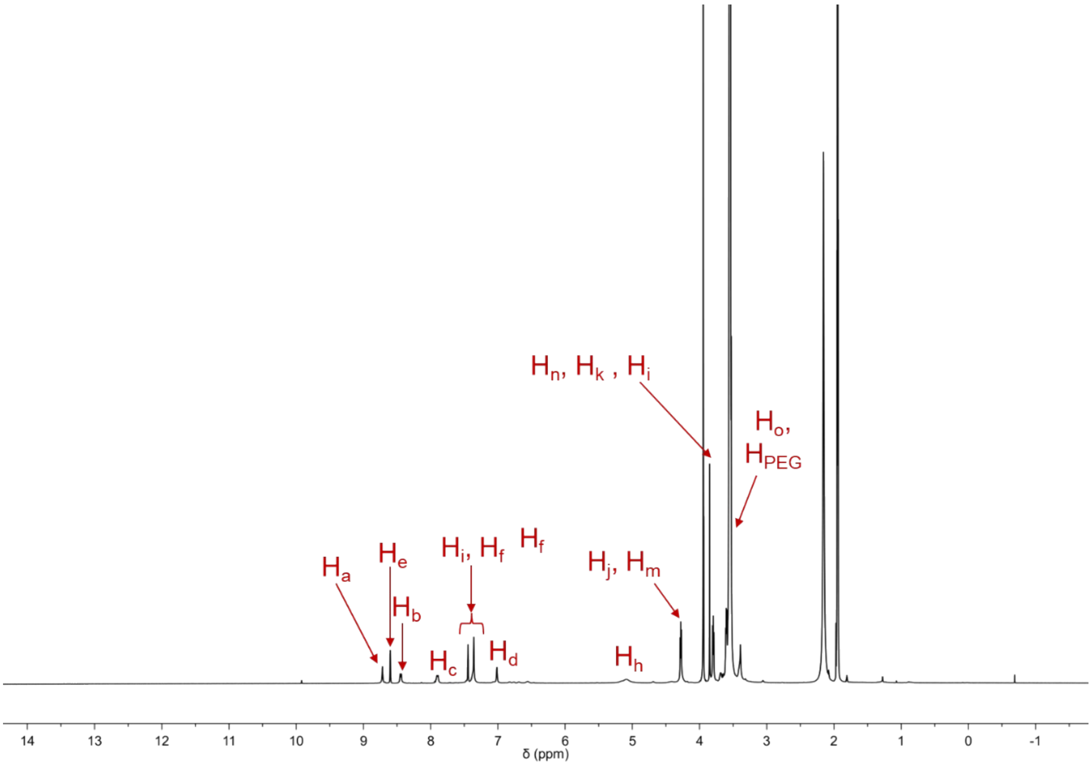

Figure S10: ^{1}H NMR spectrum (400 MHz, 298 K, CD_3CN) of cage **2**.

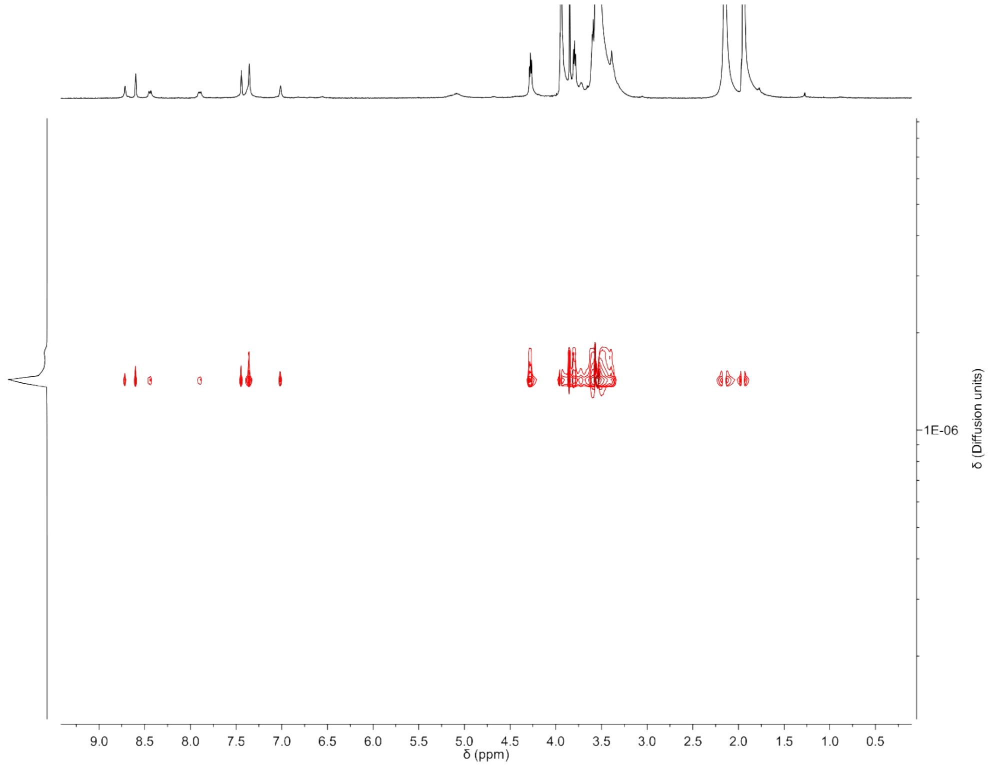

Figure S11: ^{1}H DOSY spectrum of cage **2** in CD_3CN (298 K, 400 MHz), D ~ 1.43×10^{-6} cm^2 s^{-1}.

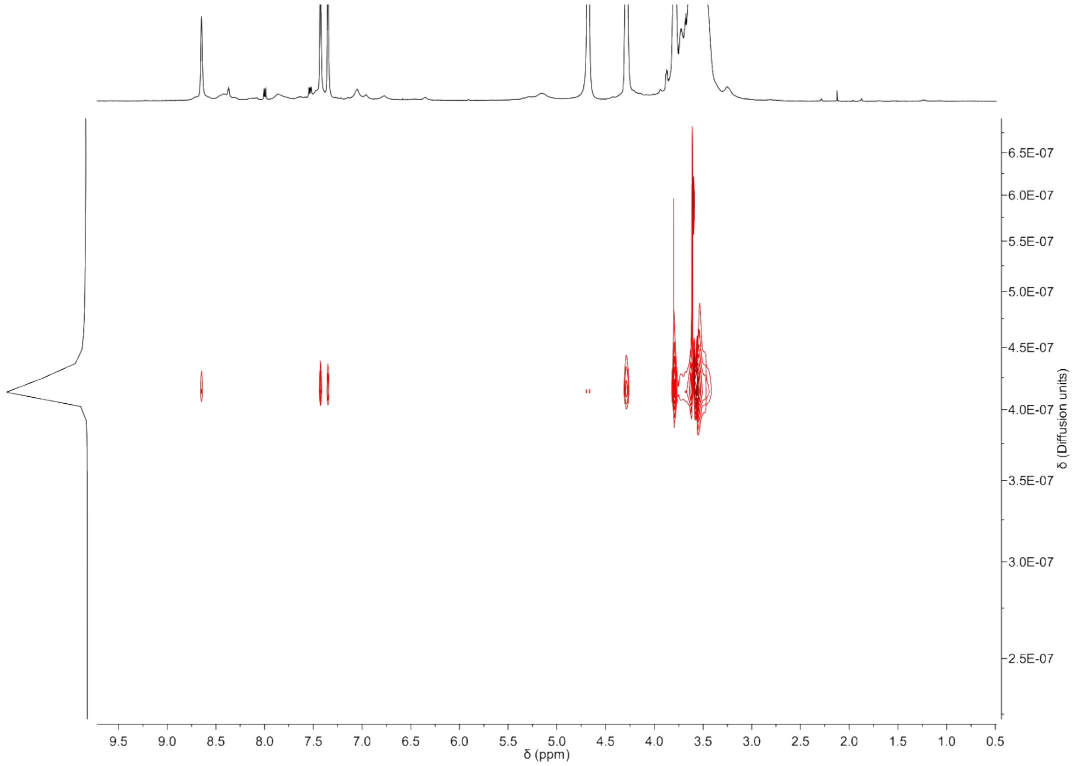

Figure S12: ^{1}H DOSY spectrum of cage **2** in D_2O (298 K, 400 MHz), D ~ 4.14×10^{-7} cm^2 s^{-1}.

2.7) Hetero-cage 1•2

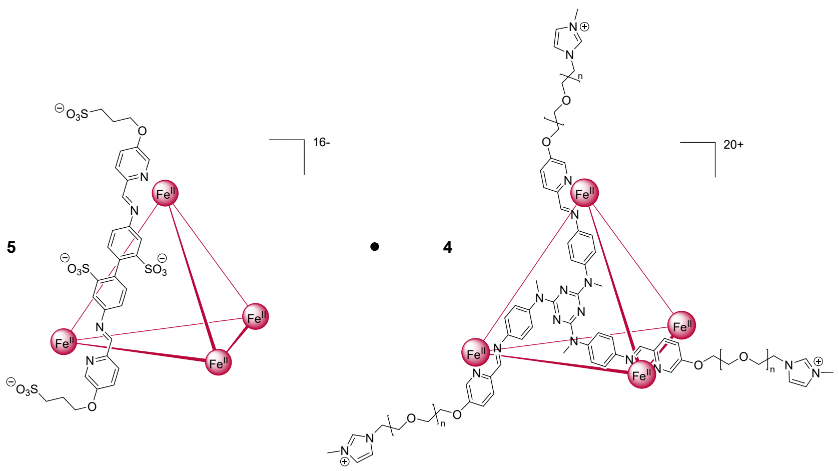

Cage **1** (5.8 mg, ~ 0.82 µmol) in water (1 mL) was added to cage **2** (16.3 mg, ~ 0.76 µmol) in methanol (1 mL) and the resulting mixture was washed with ethyl acetate (3 × 10 mL) and then dried under a stream of nitrogen gas to yield flaky red solid salt [**1**]$_5$•[**2**]$_4$ (16.4 mg, ~ 0.19 µmol, approximately quantitative). ^{1}H NMR (400 MHz, D_2O, 298 K, referenced to residual solvent at 4.79 ppm) δ_H = 9.27 (bs), 8.84 (bs), 8.75 (bs), 7.93 (bs), 7.78 (bs), 7.53 (bs), 7.44 (bs), 6.43 (bs), 5.83 (bs), 4.39 (bs), 4.14 (bs), 3.89 (bs), 3.35 – 3.75 (m), 3.06 (bs), 2.95 (bs), 2.07 (bs), 1.24 (bs). ^{13}C NMR (125 MHz, D_2O, 298 K) δ_C = 142.1, 139.4, 137.3, 136.3, 133.2, 133.0, 127.0, 123.4, 122.5, 121.5, 121.1, 118.2, 71.7, 69.5, 68.4, 60.3, 49.0, 47.5, 35.7, 30.3, 24.2, 23.8

The broad PEG signals were likely to have been caused by rapid relaxation and this hindered the collection of 2D NMR spectra. The harsh conditions required for mass spectrometry induce decomposition of the cage, thus precluding acquisition of mass spectra.

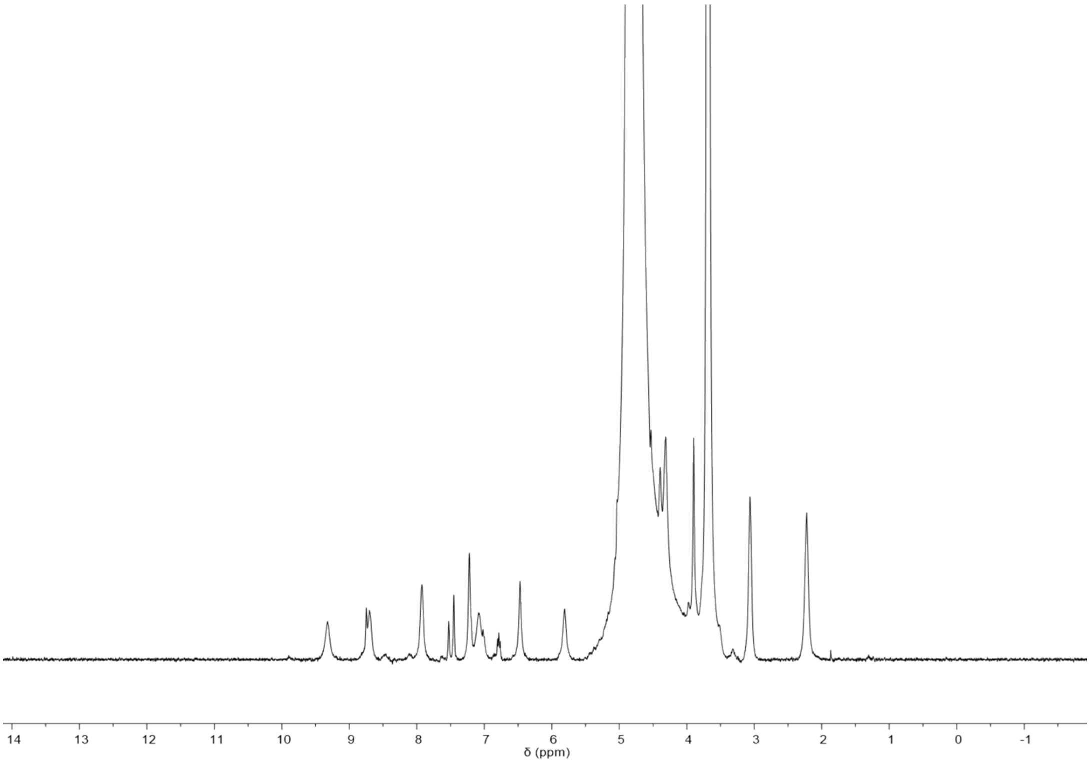

Figure S13: ^{1}H NMR spectrum (500 MHz, 298 K, D_2O) of cage **1•2**.

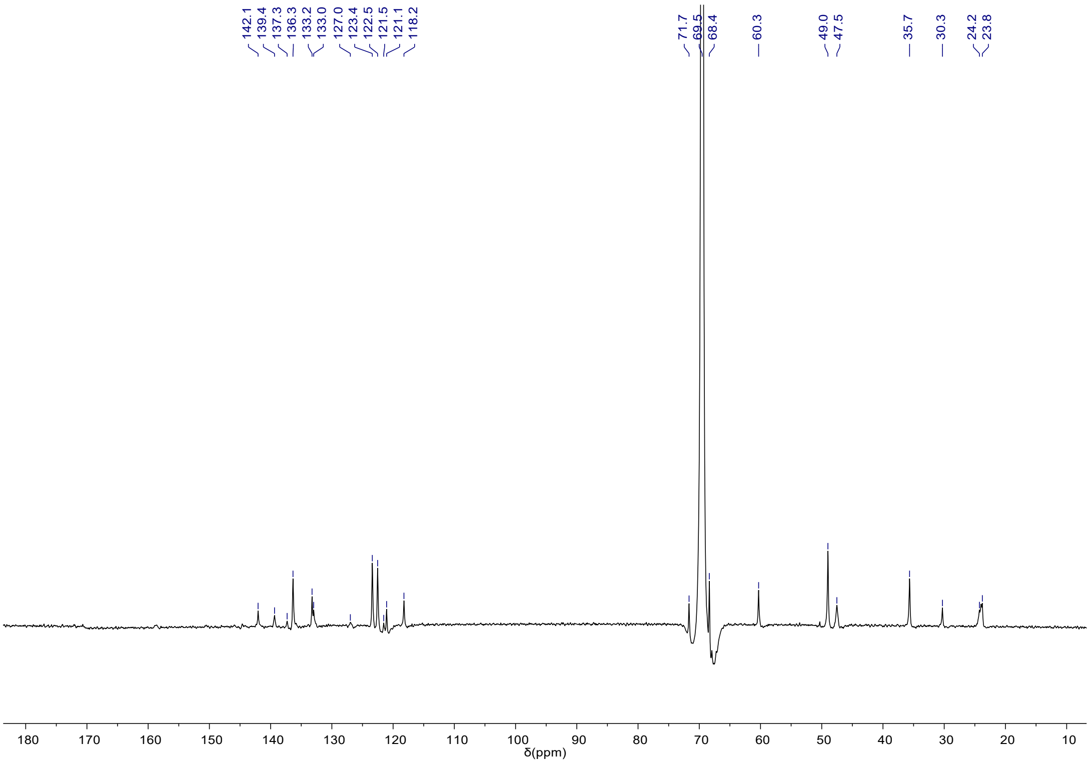

Figure S14: ^{13}C NMR spectrum (125 MHz, 298 K, D_2O) of cage **1•2**.

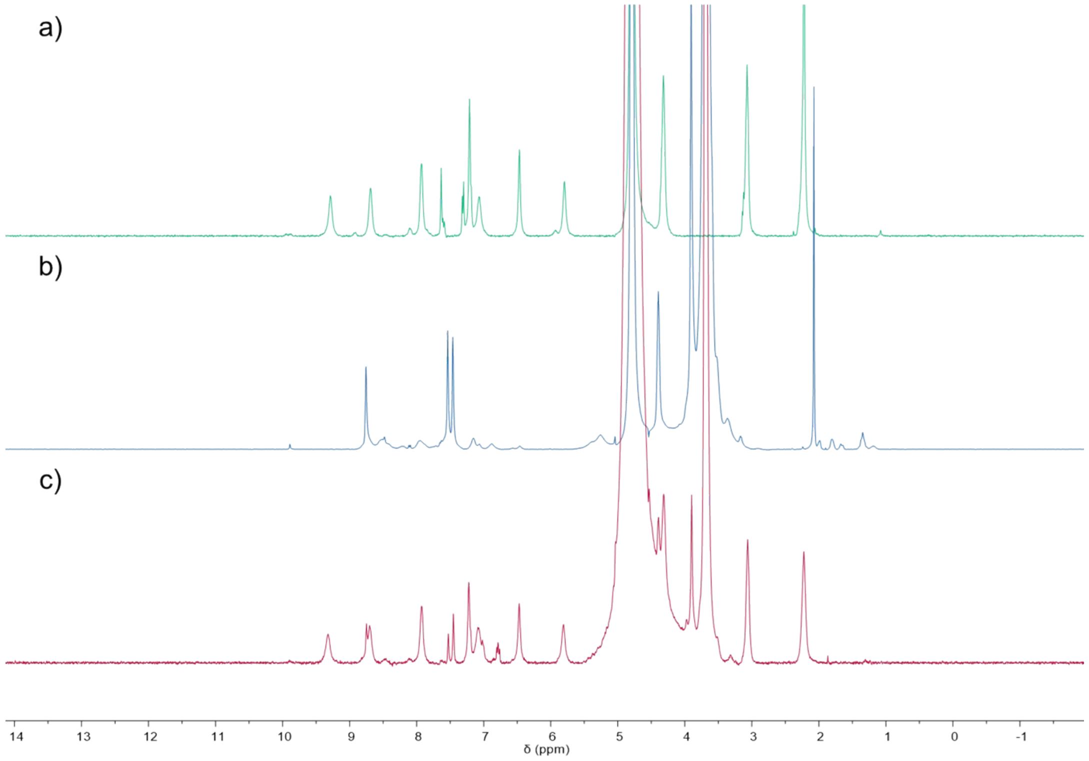

Figure S15: ^{1}H NMR spectra (500 MHz, 298 K, D_2O) of a) cage **1**, b) cage **2**, c) porous salt **1•2**.

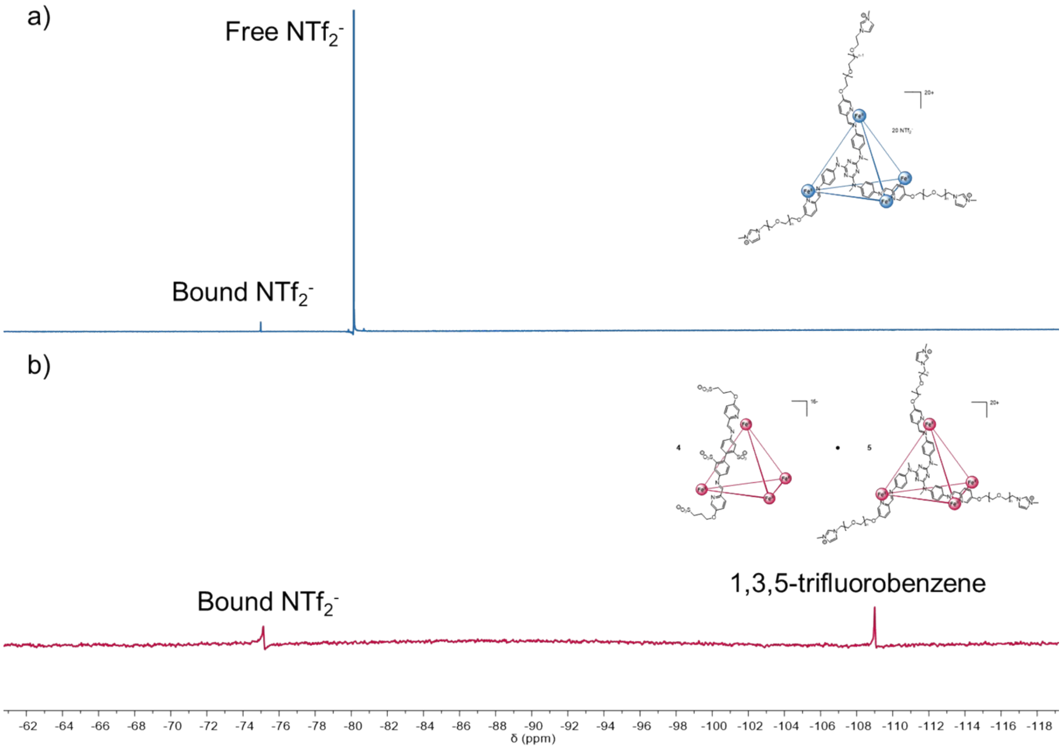

Figure S16: a) ^{19}F NMR spectra (298 K, 400 MHz, D_2O) of **2**; −75.5 ($NTf_2^- \subset$ **2**), −79.8 (free NTf_2^-), b) ^{19}F NMR spectra (298 K, 400 MHz, D_2O) for the formation of **1•2**; −75.5 ($NTf_2^- \subset$ **1•2**), −109.0 (1,3,5-trifluorobenze, reference capillary). Moreover, the signal for triflimide at −74.9 ppm, remaining in the ^{19}F NMR spectrum of **1•2** in D_2O, corresponds to triflimide bound within the cavity of cage **2**. All shifts in ppm.

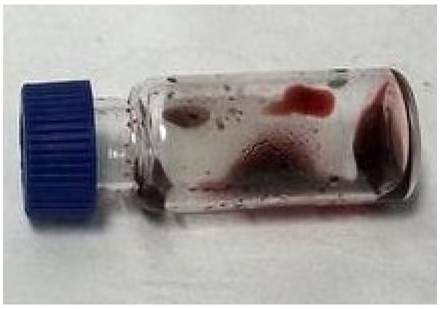

Figure S17: Photograph of a sample of ionic liquid **1•2**.

3) Single-crystal X-ray diffraction

Data were collected at Beamline I19 of Diamond Light Source employing silicon double crystal monochromated synchrotron radiation (0.6889 Å) with ω and ψ scans at 100(2) K.[8] Data integration and reduction were undertaken with Xia2.[9, 10] Subsequent computations were carried out using the WinGX-32 graphical user interface.[11] A multi-scan empirical absorption correction using spherical harmonics was applied to the data using DIALS.[10] The structure was solved by intrinsic phasing using SHELXT[12] then refined and extended with SHELXL.[13] Carbon-bound hydrogen atoms were included in idealised

positions and refined using a riding model. Disorder was modelled using standard crystallographic methods including constraints, restraints, and rigid bodies where necessary. Crystal structure images were prepared using PyMOL Molecular Graphics System, Version 1.4.1.

The crystals with composition $[(C_3H_6O)_{0.5}$□**1**$]\cdot 16K\cdot 84.5H_2O$ were grown by diffusion of acetone into an aqueous solution of the complex. The crystals employed immediately lost solvent after removal from the mother liquor. However rapid handling prior to flash cooling in liquid nitrogen and the use of synchrotron radiation enabled data to be collected to approximately 0.8 Å resolution. The asymmetric unit was found to contain one complete Fe_4L_6 assembly as well as associated counterions and solvent molecules. Bond lengths and angles within pairs of chemically identical organic ligands were restrained to be similar to each other (SAME) and thermal parameter restraints (SIMU, RIGU) were applied to all atoms except for iron and potassium. Additional DFIX and DANG restraints were applied to disordered regions of the structure.

The structure displayed a high degree of disorder. All but one of the peripheral propylsulfonate chains and several of the central sulfonate groups were modelled as disordered over two or three locations with minor occupancy groups modelled with isotropic thermal parameters. The thermal parameters of some of the minor occupancy locations of these groups remain larger than ideal and most of the remaining electron density peaks are close to the disordered sulfonate groups, consistent with further minor disorder which could not be resolved.
The potassium counterions and solvent water molecules also show very high levels of disorder. Due to this disorder only a fraction of the expected number of potassium ions could be conclusively identified. The remaining electron density was modelled as disordered water molecules. The occupancies of all located potassium ions and water molecules were refined freely and then fixed at the obtained values. No hydrogen atoms were modelled for the water molecules due to their high level of disorder. The encapsulated acetone molecule was also modelled as disordered over two locations.

CheckCIF gives numerous A and B level alerts due to the water molecules for which hydrogen atoms were not modelled (Short Inter D…A Contacts, apparent Isolated Oxygen Atoms). In addition to these alerts there are a further 2 level A alerts and 7 B level alerts. These alerts result from differences between the calculated and reported formula due to the missing hydrogens and potassium ions, unresolved disorder of the sulfonate groups (large NonSolvent Ueq(max)/Ueq(min) ranges for O and S) and the high wR2 value arising from the inability to completely model the disorder of the waters and potassium counterions. Three of the B level alerts result from positive residual density close to the iron atoms arising from absorption effects and/or beam damage from the synchrotron radiation.
Crystallographic data have been deposited with the CCDC (2177314).

Formula $C_{181.5}H_{328}Fe_4K_{16}N_{24}O_{169}S_{24}$, M 7169.10, Monoclinic, space group C 2/c (#15), *a* 49.73510(16), *b* 37.80240(8), *c* 40.72020(11) Å, *β* 125.5730(2), *V* 62270.6(3) Å^3, D_c 1.529 g cm^{-3}, *Z* 8, crystal size 0.20 by 0.15 by 0.10 mm, colour purple, habit block, temperature 100(2) Kelvin, *λ*(Synchrotron) 0.6889 Å, *μ*(Synchrotron) 0.604 mm^{-1}, $T(\text{Analytical})_{min,max}$ 0.958637417924, 1.0, $2\theta_{max}$ 51.01, *hkl* range -54 62, -47 47, -50 50, N 320873, N_{ind} 63348(R_{merge} 0.0549), N_{obs} 40633(I > 2σ(I)), N_{var} 4373, residuals* *R*1(*F*) 0.1216, *wR2*(F^2) 0.3577, GoF(all) 1.221, $\Delta\rho_{min,max}$ -1.021, 1.629 e$^-$ Å^{-3}.

*R1 = $\Sigma||F_o| - |F_c||/ \Sigma|F_o|$ for $F_o > 2\sigma(F_o)$; $wR2 = (\Sigma w(F_o^2 - F_c^2)^2/ \Sigma(wF_c^2)^2)^{1/2}$ all reflections
w=1/[$\sigma^2(F_o^2)$+(0.2000P)^2+75.0000P] where P= ($F_o^2 + 2F_c^2)/_3$

In order to determine the available cavity volume within the cages, MoloVol calculations based on the crystal structure were performed (Figure S15).[14] A virtual probe with a radius of 1.2 Å was employed with a grid resolution of 0.1 Å and optimization depth of 4. All other parameters were set at their default values. A volume of 127.3 Å^3 was obtained for cage **1**, within the range of reported volumes (115-141 Å^3) for crystal structures of the analogous cage prepared from 2-formylpyridine.[15-17] Using the same method, the volume of the previously reported $Fe^{II}_4L_4$ cage prepared from the same central sub-component as **2** was calculated to be 228.3 Å^3, similar to the reported value of 233 ± 2 Å^3.[6]
Considering just the central cavity, the free space per gram for the two cages **1**, **2** and the porous salt **1•2** was calculated by using the previously reported equation (2):[18]

$$V_{free} = \frac{V \times h \times m}{M} \quad (2)$$

V = volume of cage (Å^3), h = Avogadro's constant, m = mass of cage (g) and M = molar mass of cage (g mol^{-1}).
The free space per gram for cage **2** was calculated using the volume of the previously reported $Fe^{II}_4L_4$ cage, and a molar mass (M) of 24436.48 g/mol (assuming an average of 27 $-CH_2CH_2O-$ repeat units per PEG chain).
The free space for gram for hetero cage **1•2** was calculated considering the ratio of **1**: **2** of 5:4, and a molar mass (M) of 125834.3 g/mol.

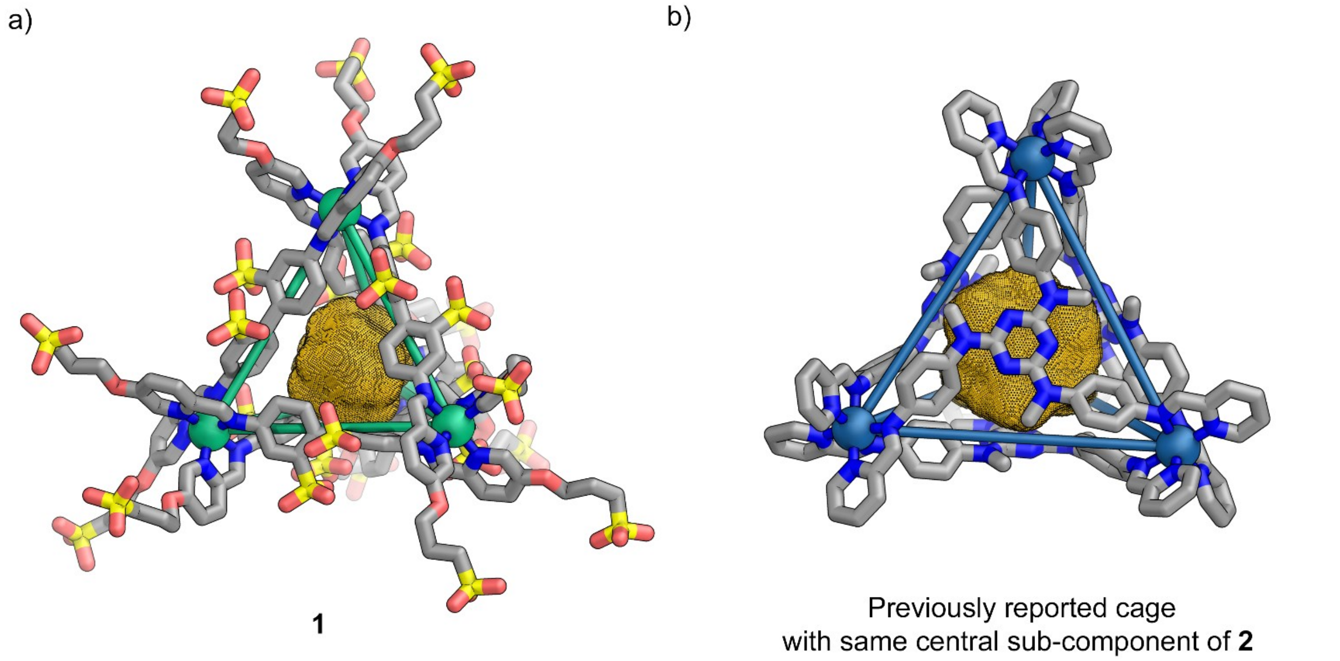

Figure S18: MoloVol-calculated void space within the cavity of a) cage **1** and b) previously reported cage with the same central sub-component of **2**[6].

4) TEM-EDS

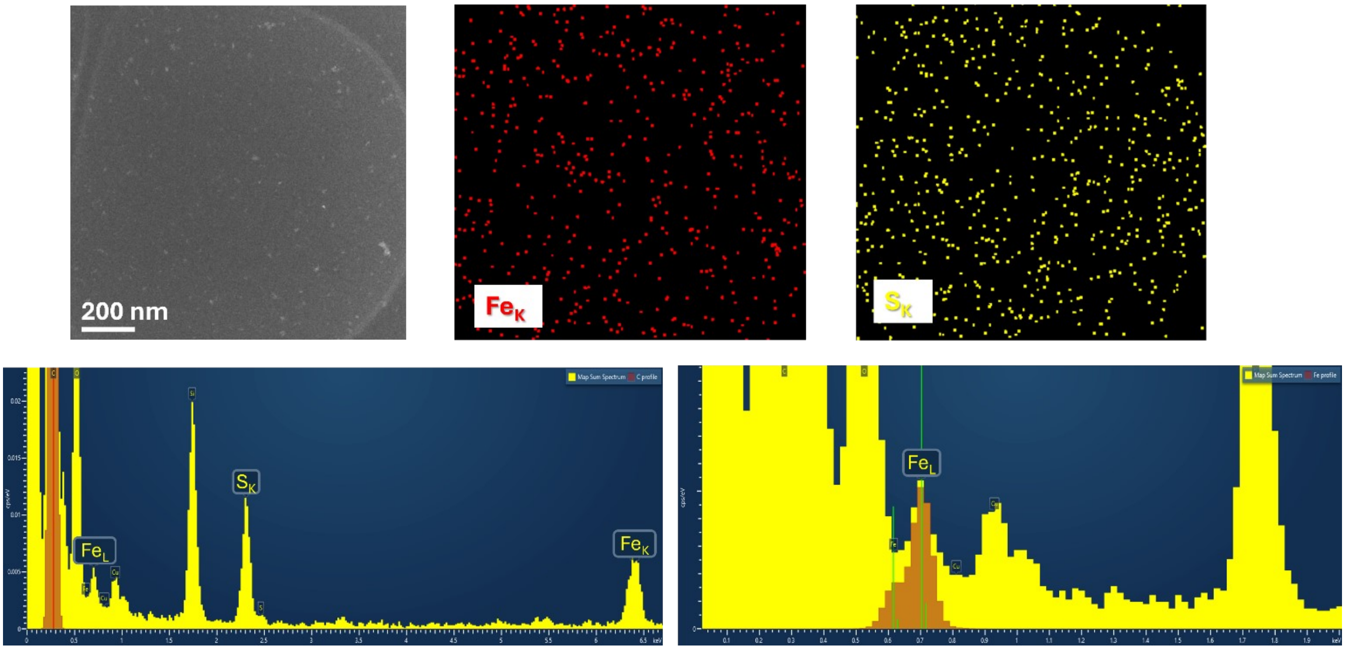

Figure S19: Top panel: STEM–HAADF image of **1•2** showing a continuous film crossing a hole of the holey carbon support, together with the corresponding EDS elemental maps of Fe and S. Bottom panels: EDS spectrum and a magnified view of the Fe$_L$ peak, whose spectral shape can only be reproduced by accounting for the presence of Fe and the absence of F. No signals from F or K were detected, consistent with the absence of NTf_2^- and K^+ counterions.

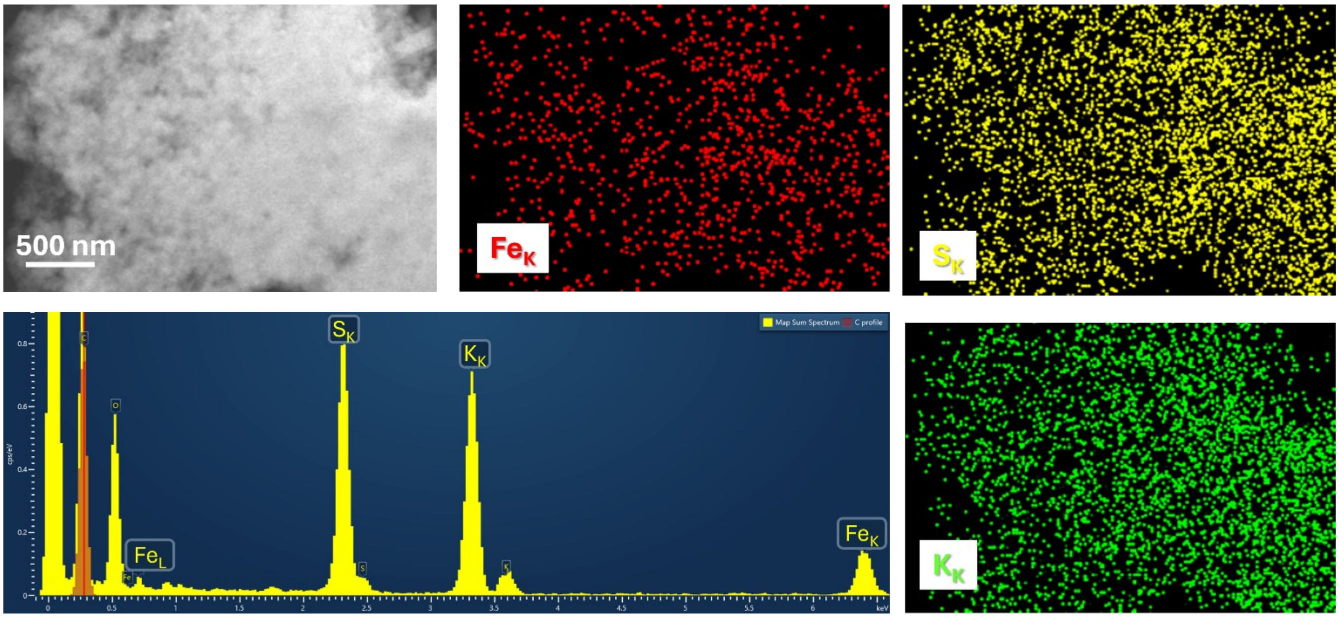

Figure S20: STEM-HAADF image of **1**. Right: corresponding EDS spectrum and elemental maps of Fe, S and K.

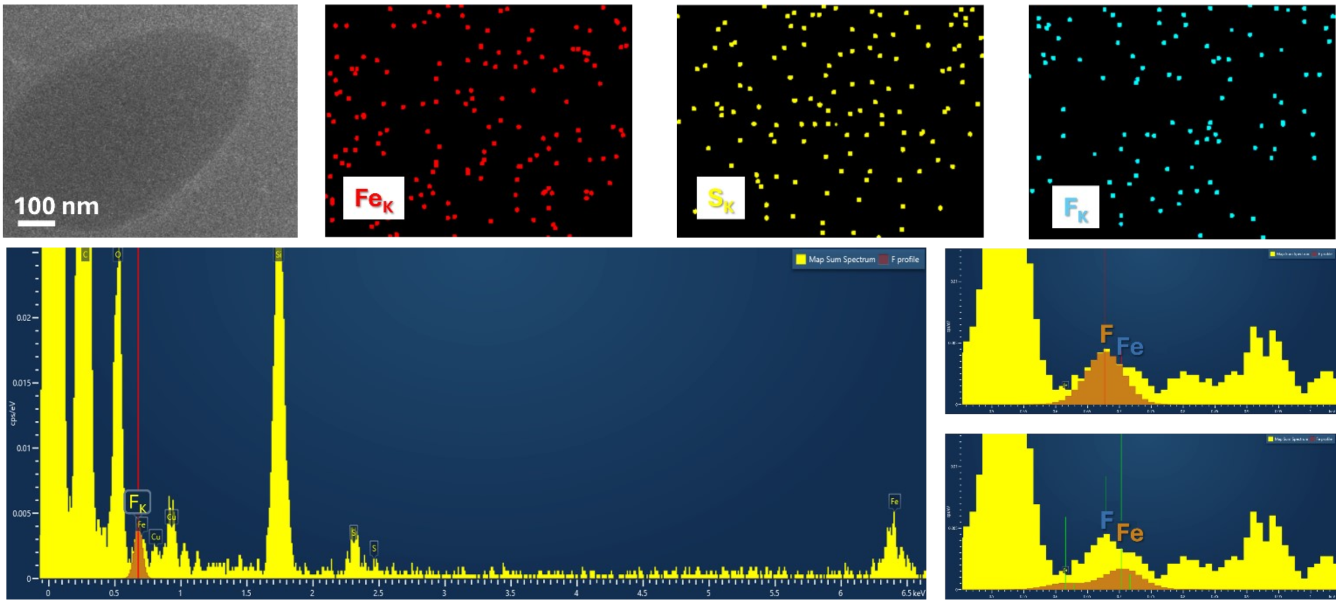

Figure S21: STEM-HAADF image of **2**, showing a continuous film spanning a hole of the holey carbon support, together with the corresponding EDS elemental maps of Fe, S, and F. The bottom panels show the EDS spectrum and two magnified views of the same experimental EDS peak, whose spectral shape can only be reproduced by accounting for the simultaneous presence of Fe and F, consistent with the presence of NTf_2^- counterions.

5) Host-guest chemistry in solution

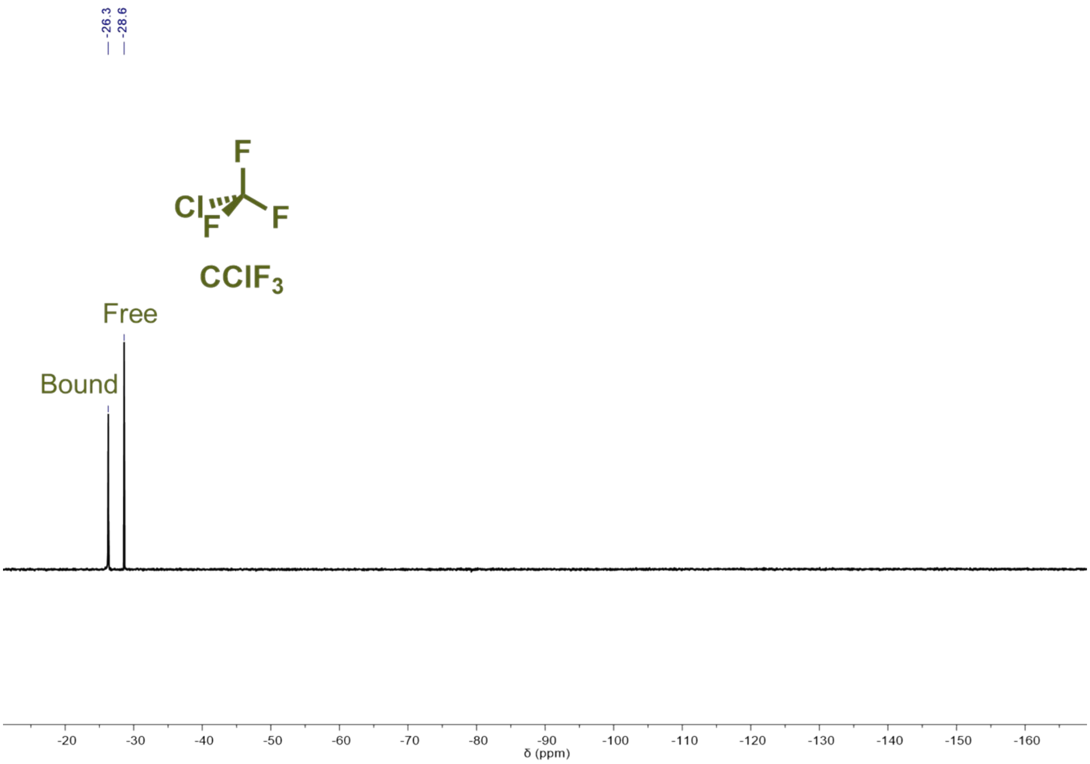

Figure S22: ^{19}F NMR spectra (298 K, 400 MHz, D_2O) of **1** with $CClF_3$; −26.3 ($CClF_3 \subset$ **1**), −28.6 (free $CClF_3$). All shifts in ppm.

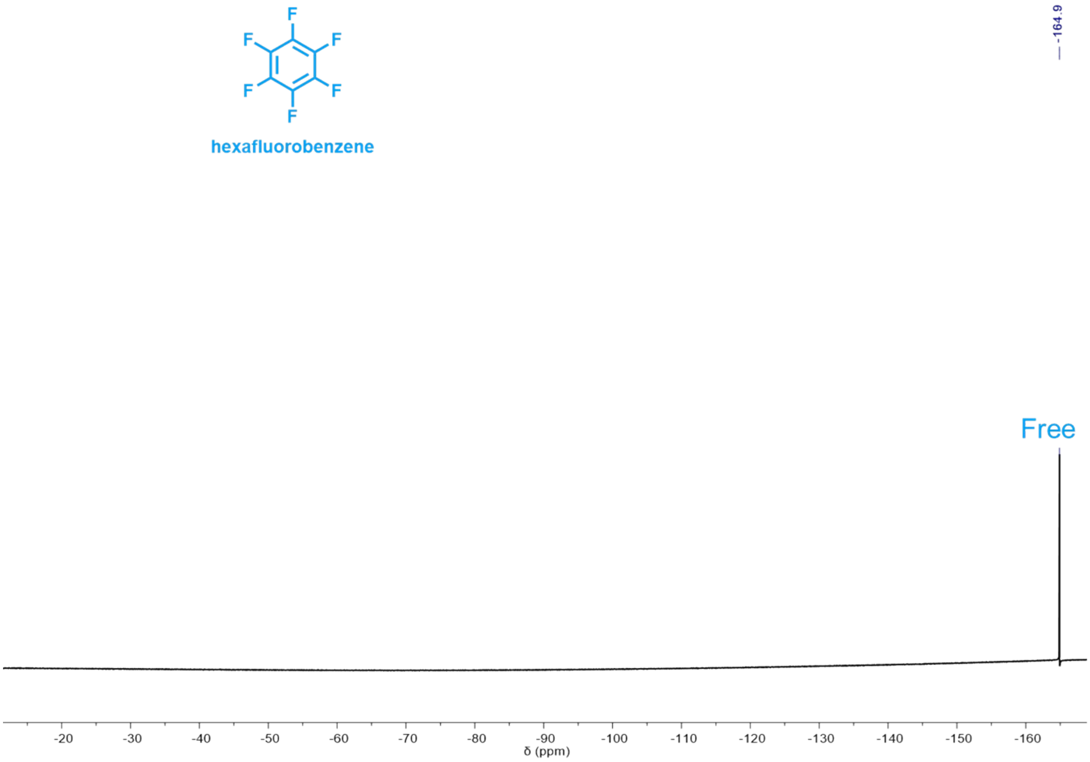

Figure S23: ^{19}F NMR spectra (298 K, 400 MHz, D_2O) of **1** with hexaflurobenzene; −164.9 (free hexafluorobenzene). All shifts in ppm.

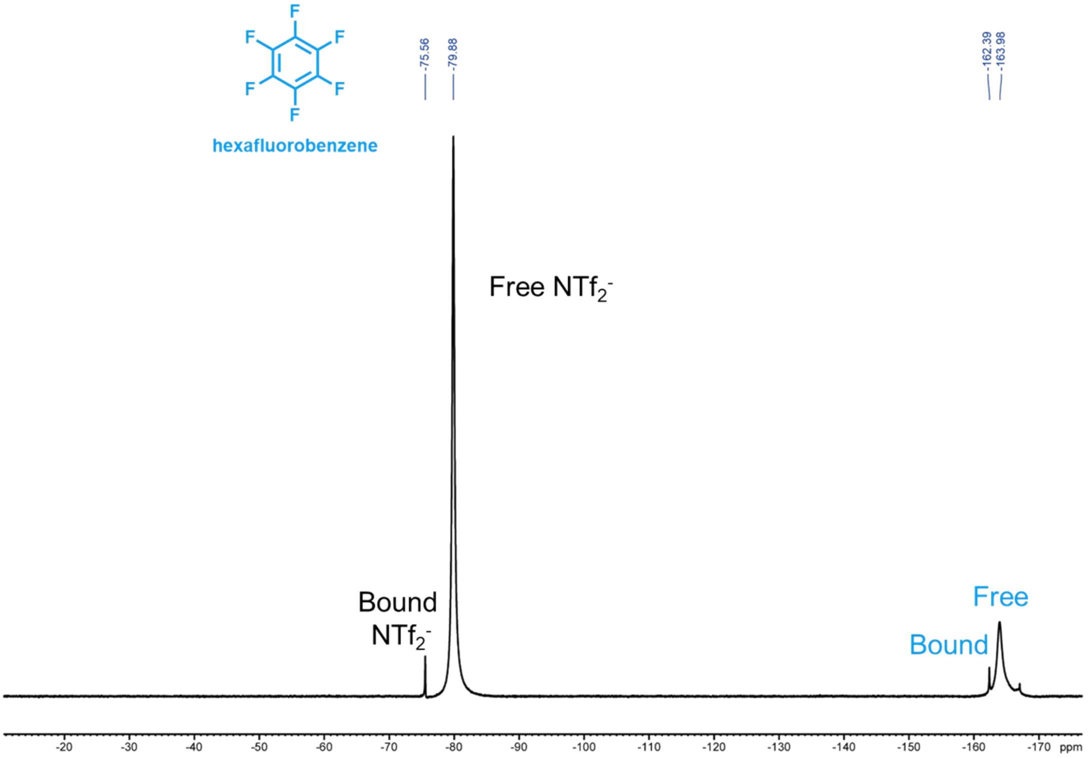

Figure S24: ^{19}F NMR spectra (298 K, 400 MHz, D_2O) of **2** with hexaflurobenzene; −75.5 ($NTf_2^- \subset$ **2**), −79.8 (free NTf_2^-), −162.3 (hexafluorobenzene ⊂ **2**), −164.0 (free hexafluorobenzene). All shifts in ppm.

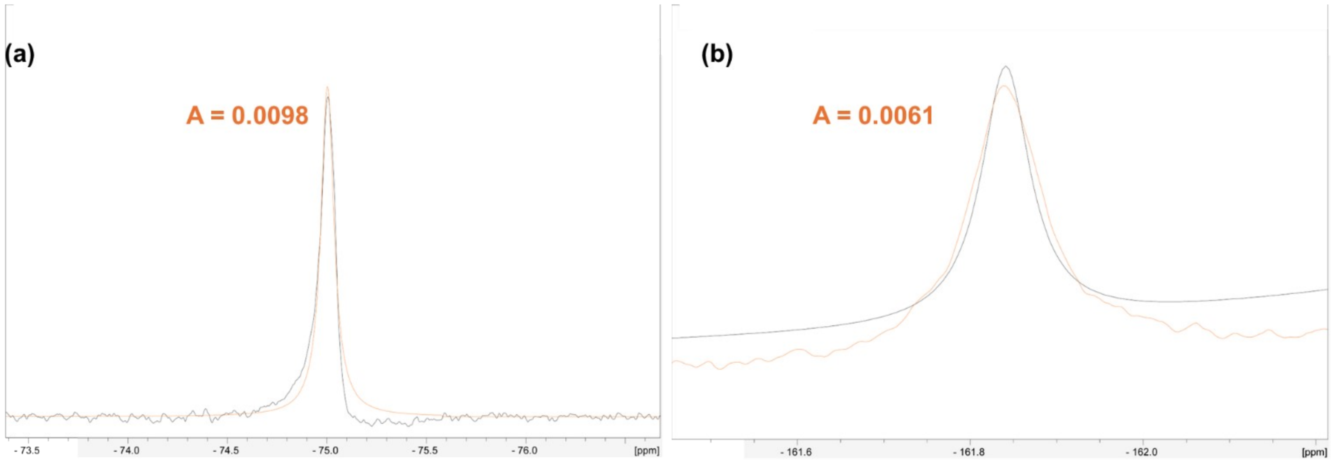

Figure S25: ^{19}F NMR spectra (298 K, 400 MHz, D_2O) of **2** host-guest complexes (black line) and Gaussian deconvolution (orange line). (a) peak of $NTf_2^- \subset$ **2** with a deconvoluted area of 0.0098. (b) peak of hexafluorobenzene ⊂ **2** with a deconvoluted area of 0.0061. The percentage of bound hexafluorobenzene relative to bound NTf_2^- is 38 %.

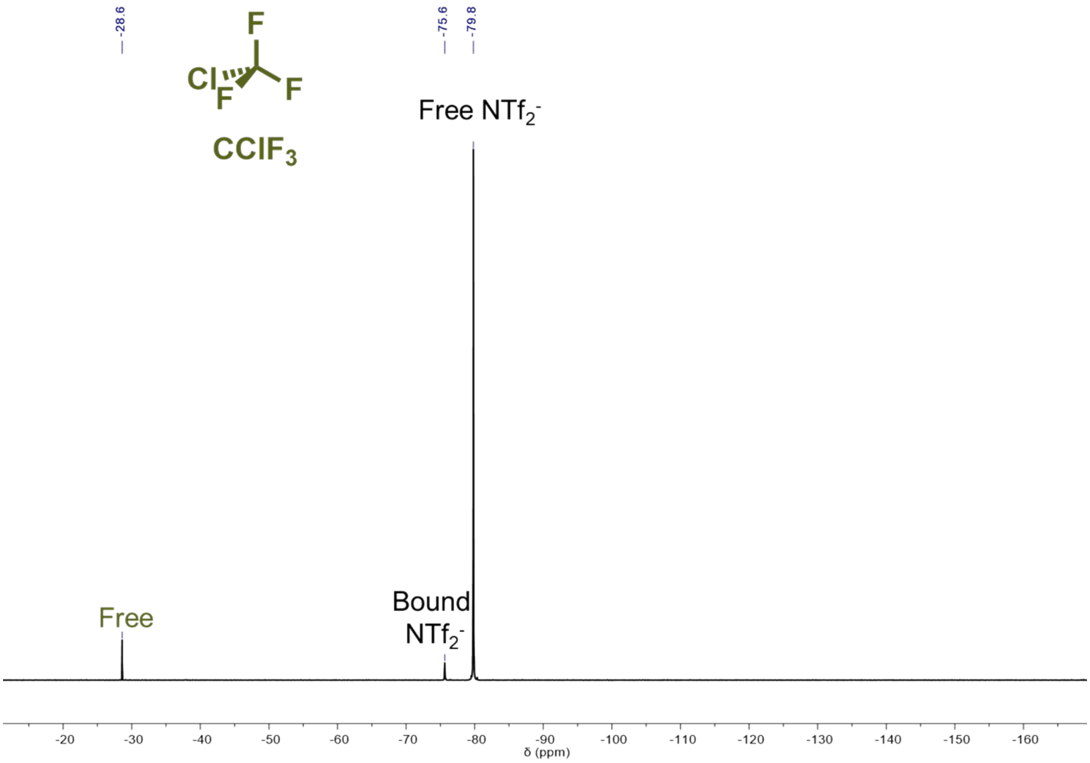

Figure S26: ^{19}F NMR spectra (298 K, 400 MHz, D_2O) of **2** with $CClF_3$; −28.6 (free $CClF_3$), −75.6 (NTf_2^- ⊂ **2**), −79.8 (free NTf_2^-). All shifts in ppm.

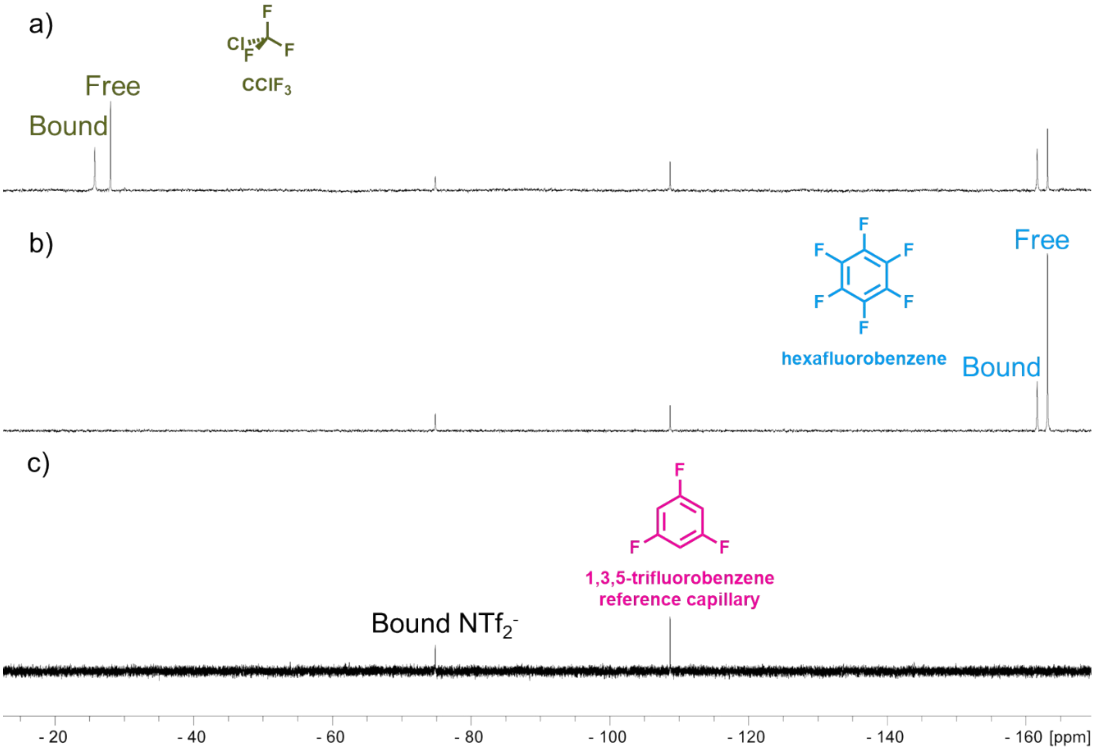

Figure S27: ^{19}F NMR spectra (298 K, 400 MHz, D_2O) of **1•2** host-guest complexes. a) hexafluorobenzene and $CClF_3$; −26.3 ($CClF_3$ ⊂ **1•2**), −28.6 (free $CClF_3$). −75.5 (NTf_2^- ⊂ **1•2**), −109.0 (1,3,5-trifluorobenze, reference capillary), −162.3 (hexafluorobenzene ⊂ **1•2**), −164.0 (free hexafluorobenzene). b) hexafluorobenzene; −75.5 (NTf_2^- ⊂ **1•2**), −109.0 (1,3,5-trifluorobenze, reference

capillary), −162.3 (hexafluorobenzene ⊂ **1•2**), −164.0 (free hexafluorobenzene). c) no added guests, free cage; −75.5 (NTf_2^- ⊂ **1•2**), −109.0 (1,3,5-trifluorobenze, reference capillary). All shifts in ppm.

1

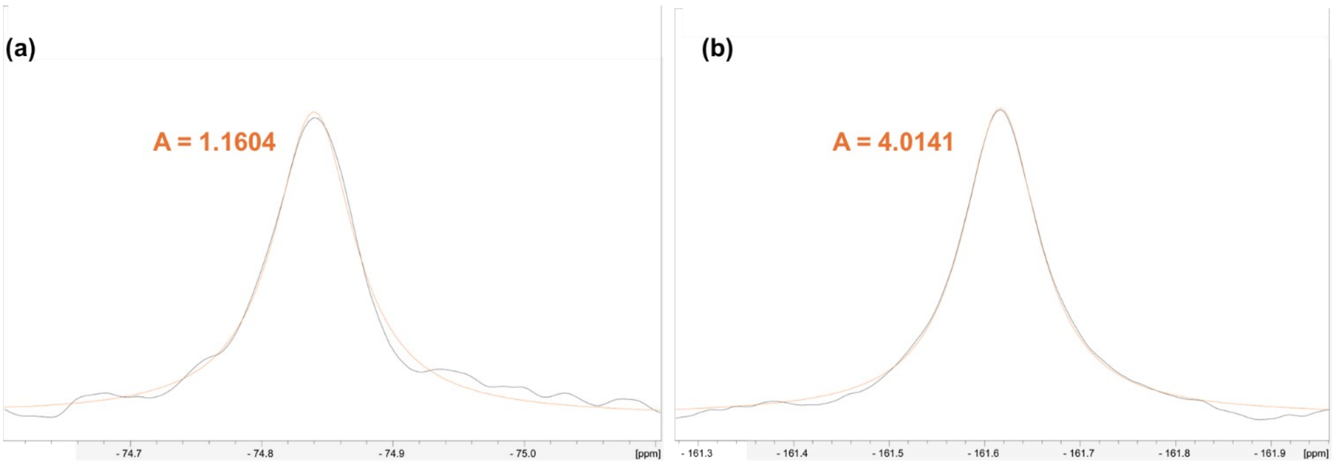

Figure S28: ^{19}F NMR spectra (298 K, 400 MHz, D_2O) of **1•2** host-guest complexes (black line) and Gaussian deconvolution (orange line). (a) peak of NTf_2^- ⊂ **1•2** with a deconvoluted area of 1.1604. (b) peak of hexafluorobenzene ⊂ **1•2** with a deconvoluted area of 4.0141. The percentage of bound hexafluorobenzene relative to bound NTf_2^- is 78 %.

The relative percentage of encapsulated hexafluorobenzene within **1•2** was estimated based on the deconvoluted peak areas obtained from ^{19}F NMR. The area corresponding to encapsulated hexafluorobenzene was divided by the sum of the areas of hexafluorobenzene and NTf_2^- peaks (Equation 3). This provided an estimation of the relative amount of guest encapsulated versus the counterion present.

$$\%_{hexafluorobenzene\ \subset} = \frac{A_{hexafluorobenzene\ \subset}}{A_{hexafluorobenzene\ \subset} + A_{NTf_2^-\ \subset}} \quad (3)$$

6) Host-guest chemistry in neat state

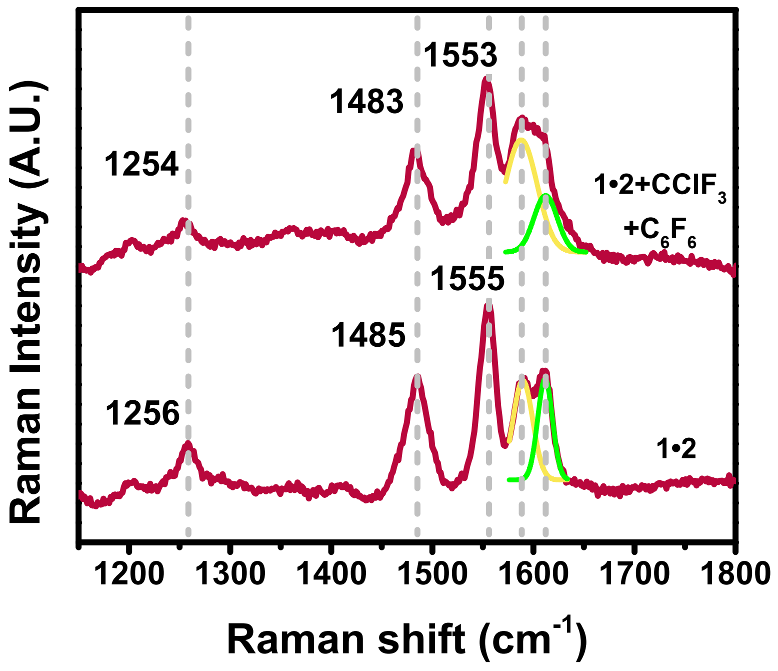

Figure S29: Raman spectra of cage **1•2** before (lower trace) and after (upper trace) encapsulation of $CClF_3$ + hexafluorobenzene, showing a 2 cm^{-1} red-shift of the C=N and N-Fe stretch from 1555 to 1553 cm^{-1}. Gaussian deconvolution of the ~1600 cm^{-1} band into two fixed-frequency sub-components (yellow and green) reveals that, although the band positions remain unchanged, the relative intensity of the two sub-components changes upon guest binding-direct evidence of host–guest interaction.

7) Thermogravimetric analysis

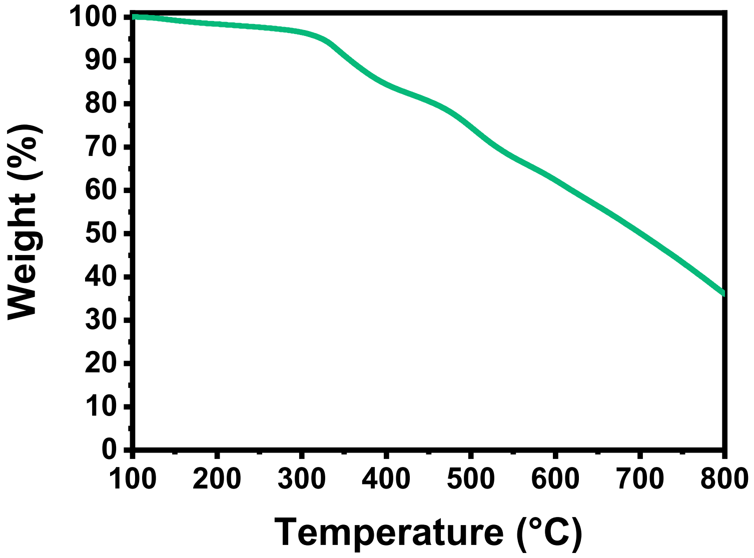

Figure S30: TGA profile for **1**.

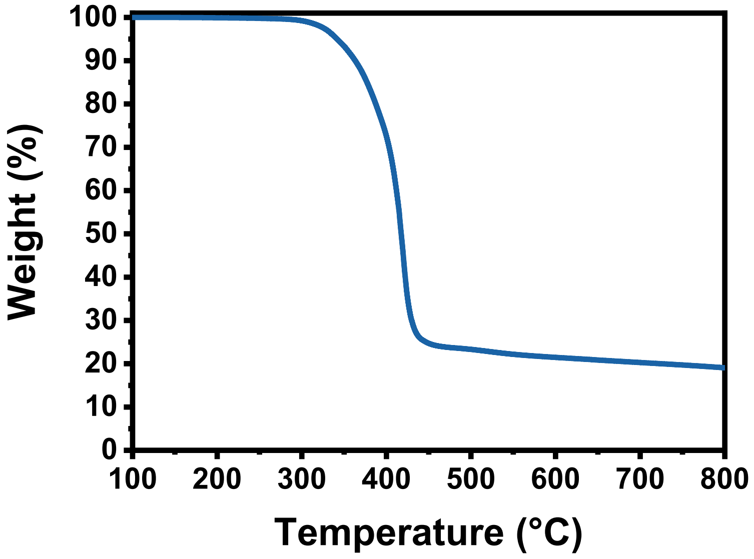

Figure S31: TGA profile for **2**.

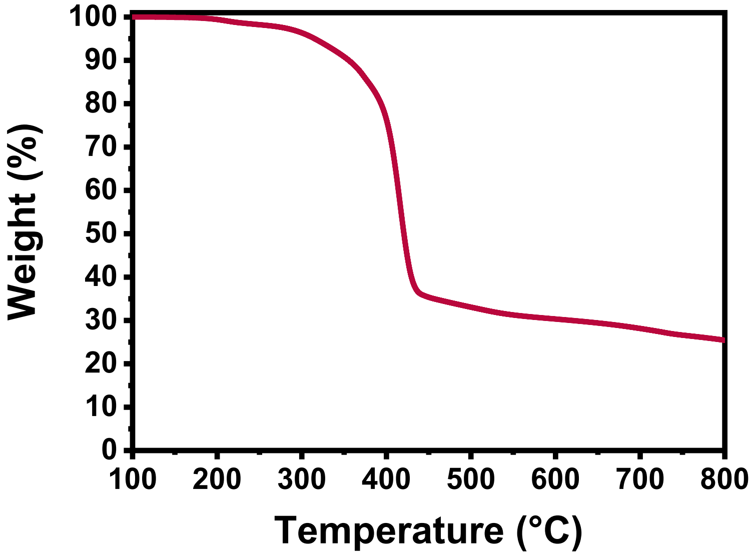

Figure S32: TGA profile for hetero-cage **1•2**.

8) **Differential Scanning Calorimetry (DSC)**

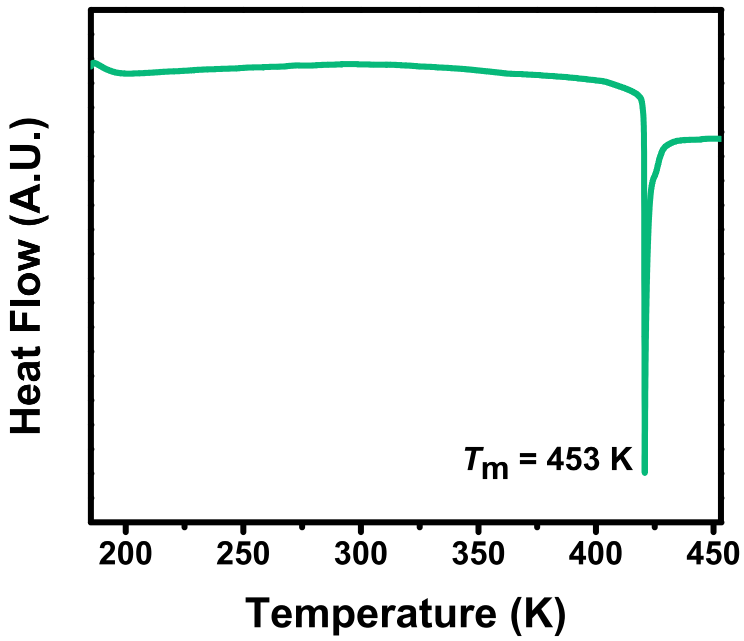

Figure S33: DSC profile for cage **1**, T_m = 453 K.

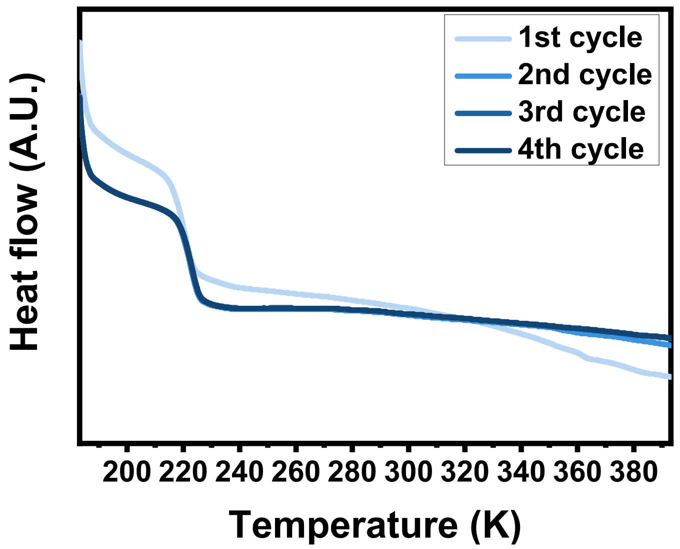

Figure S34: DSC profile for hetero-cage **2** performed for four different cycles. The first cycle differed from the following three and the 2nd one was considered reproducible for the determination of T_g.

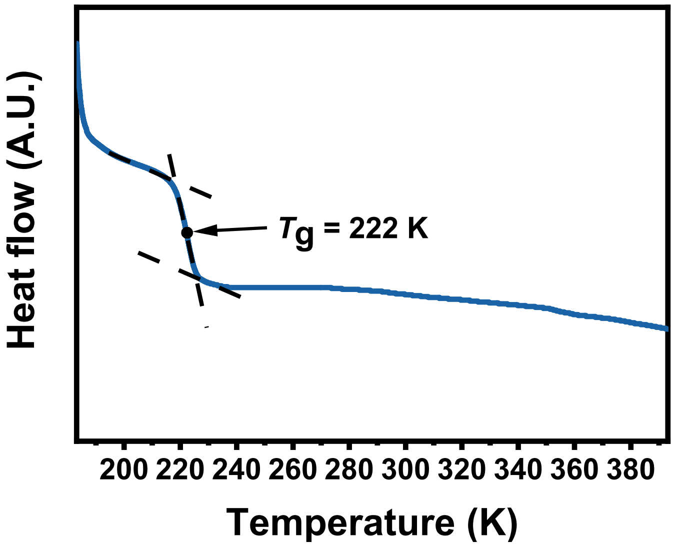

Figure S35: DSC profile (2nd cycle) of cage **2**, T_g = 222 K.

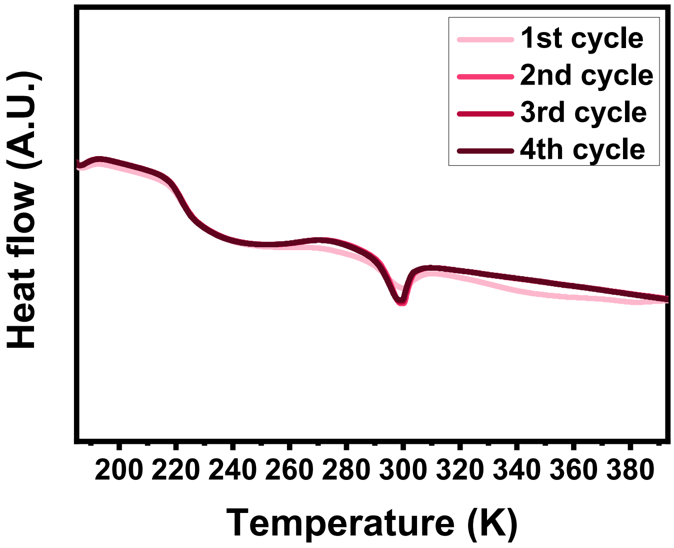

Figure S36: DSC profile for hetero-cage **1•2** performed for four different cycles. The first cycle differed from the following three and the 2nd one was considered reproducible for the determination of T_g and T_m.

9) Oscillatory rheometry

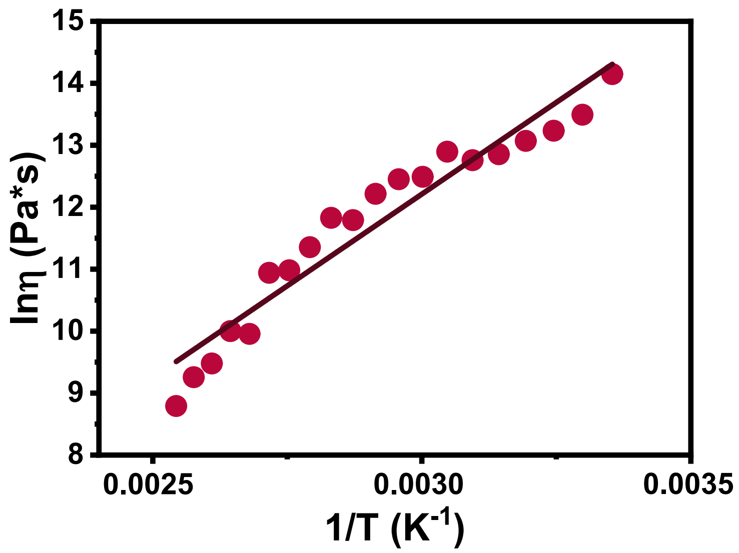

Figure S37: Downward curvature in the Arrhenius plot for hetero cage **1•2** ($r^2 < 0.98$).

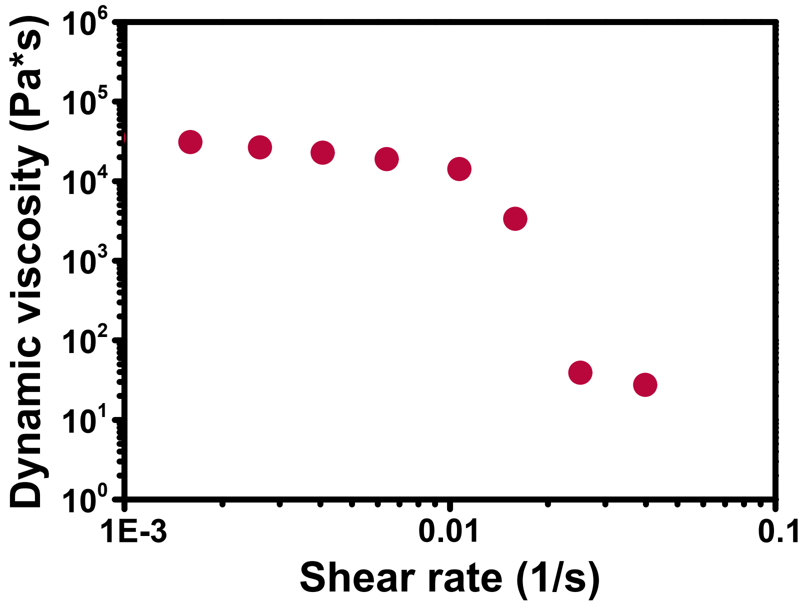

Figure S38: Shear dependent rheology; increasing in shear rate on hetero-cage **1•2** caused a drop in the viscosity of the material on account of the mechanical disruption of the structured fluid.

10) Attenuated total reflectance infrared Spectroscopy

The band at 840 cm^{-1}, absent in the spectrum of **1**, was attributed to the out-of-plane $^{+}$C(2)-H and $^{+}$C(4,5)-H bending mode with a larger motion of $^{+}$C(2)-H. The two bands at 2870 cm^{-1} and 1095 cm^{-1} were assigned to the C-H and C-O-C stretching of the PEG chains, respectively.

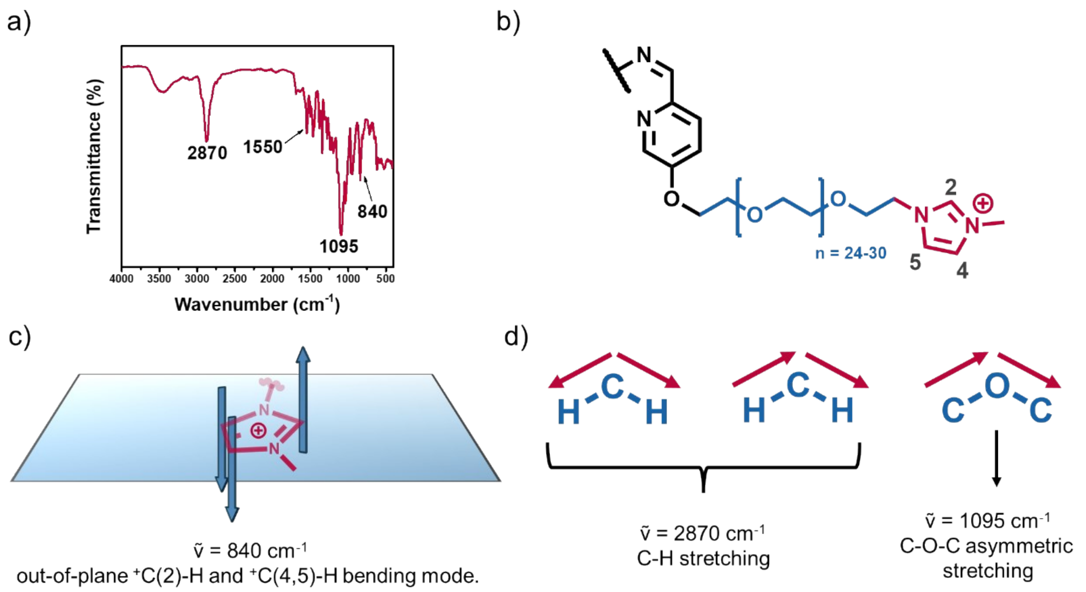

Figure S39: a) ATR of cage salt **1•2**. b) (PEG)-imidazolium chains at the vertices of the cationic cage. c) Band at 840 cm^{-1} attributed to the out-of-plane $^{+}$C(2)-H and $^{+}$C(4,5)-H bending mode. d) Bands at 2870 cm^{-1} and 1095 cm^{-1} were assigned to the C-H and C-O-C stretching of the PEG chains, respectively.

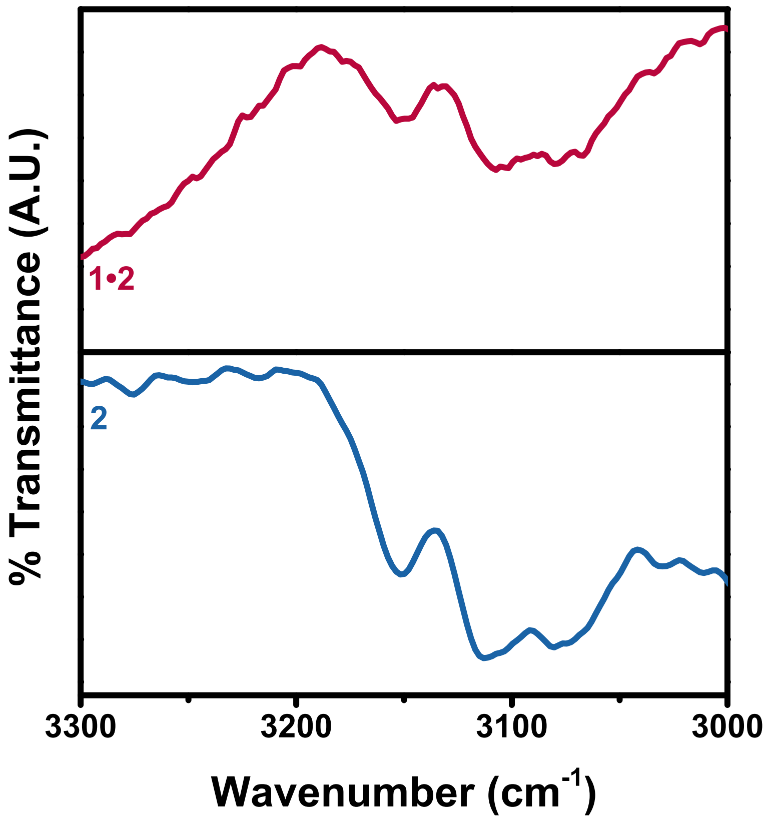

Figure S40: Stacked ATR-IR spectra of cage **2** and cage salt **1•2** in the region between 3300 and 3000 cm^{-1}. The bands were attributed to the stretching modes of the $^{+}$C(2)-H and $^{+}$C(4,5)-H groups, and the red shift observed in **1•2** indicated a change in the counterion from NTf_2^- to RSO_3^- in the cage salt.

11) **Raman Spectroscopy**

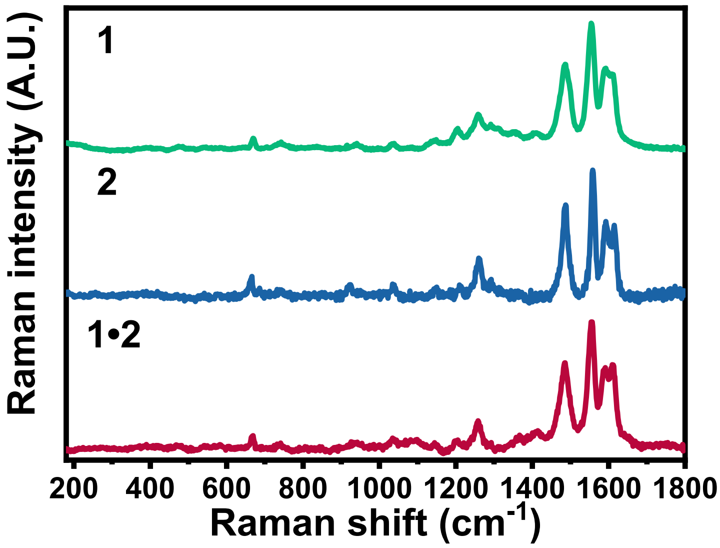

Figure S41: Visible Raman characterization of the three MOCs. Intense bands in the range between 1450 and 1650 cm^{-1} were attributed to C=N and N-Fe stretching.

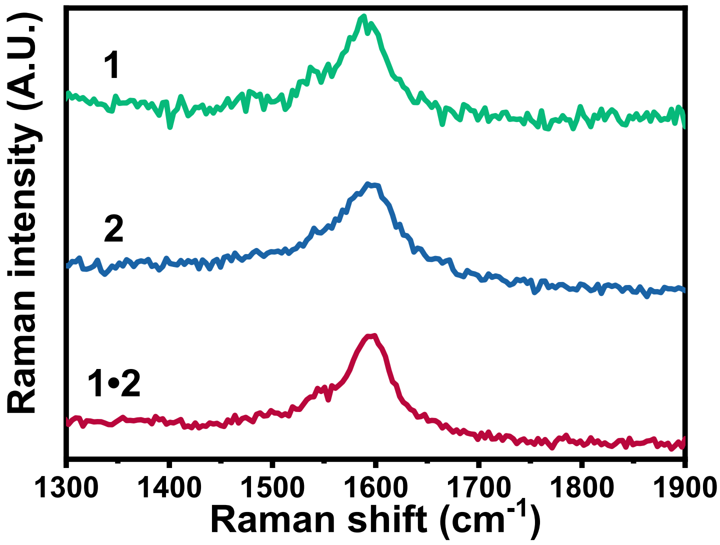

Figure S42: Resonance Raman spectra (excitation wavelength at 248 nm) of the three MOCs at 298 K. The three cages showed the similar features in the aromatic region.

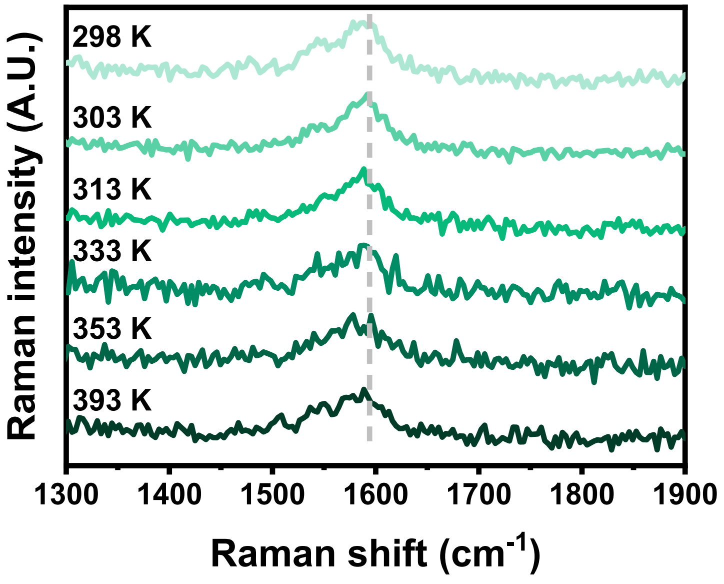

Figure S43: Resonance Raman spectra of hetero cage **1** at excitation wavelength of 248 nm at different temperatures (298 K, 303 K, 313 K, 333 K, 353 K, and 393 K).

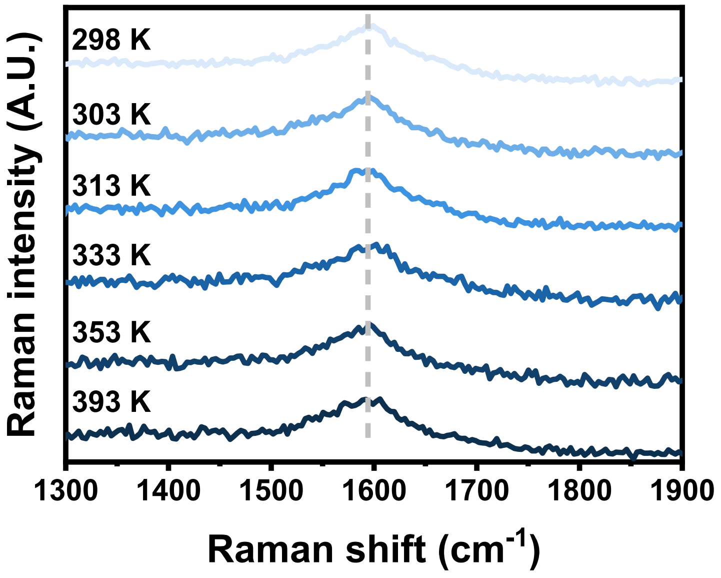

Figure S44: Resonance Raman spectra of hetero cage **2** at excitation wavelength of 248 nm at different temperatures (298 K, 303 K, 313 K, 333 K, 353 K, and 393 K).

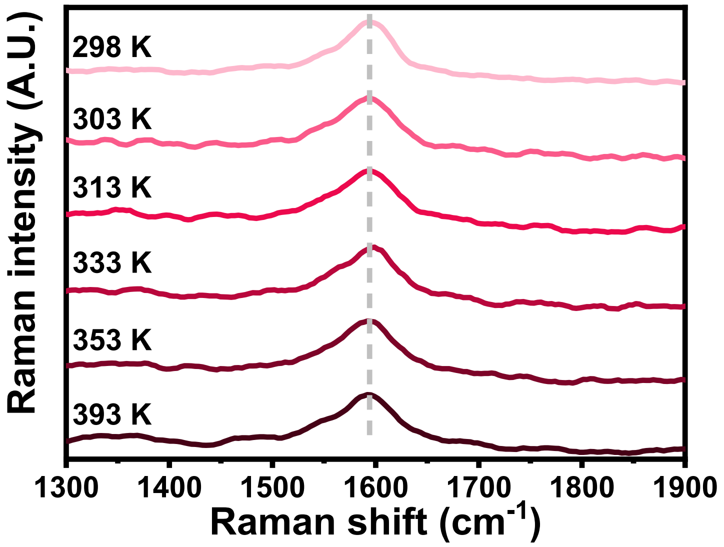

Figure S45: Resonance Raman spectra of hetero cage **1•2** at excitation wavelength of 248 nm at different temperatures (298 K, 303 K, 313 K, 333 K, 353 K, and 393 K).

References:

1. D. H. Wu, A. D. Chen, C. S. Johnson, *J. Magn. Reson., Ser. A* 1995, **115**, 260.
2. B. Bittner, R. J. Wrobel, E. Milchert, *J. Chem. Thermodyn.* 2012, **55**, 159.
3. Q. Gao, Z. Jian, *Mater. Chem. Phys.* 2020, **252**, 123252.
4. J. A. Smith, G. B. Webber, G. G. Warr, R. Atkin, *J. Phys. Chem. B* 2013, **117**, 13930.
5. H. P. Ryan, C. J. E. Haynes, A. Smith, A. B. Grommet, J. R. Nitschke, *Adv. Mater.* 2021, **33**, 2004192.
6. J. L. Bolliger, T. K. Ronson, M. Ogawa, J. R. Nitschke, *J. Am. Chem. Soc.* 2014, **136**, 14545.
7. B.-N. T. Nguyen, A. B. Grommet, A. Tron, M. C. A. Georges, J. R. Nitschke, *Adv. Mater.* 2020, **32**, 1907241.
8. D. R. Allan, H. Nowell, S. A. Barnett, M. R. Warren, A. Wilcox, J. Christensen, L. K. Saunders, A. Peach, M. T. Hooper, L. Zaja, et al., *Crystals* 2017, **7**, 336.
9. G. Winter, *J. Appl. Crystallogr.* 2010, **43**, 186.
10. G. Winter, D. G. Waterman, J. M. Parkhurst, A. S. Brewster, R. J. Gildea, M. Gerstel, L. Fuentes-Montero, M. Vollmar, T. Michels-Clark, I. D. Young, et al., *Acta Crystallogr. D Struct. Biol.* 2018, **74**, 85.
11. L. Farrugia, *J. Appl. Crystallogr.* 2012, **45**, 849.
12. G. Sheldrick, *Acta Crystallogr. A* 2015, **71**, 3.
13. G. M. Sheldrick, *Acta Crystallogr. C* 2015, **71**, 3.
14. J. B. Maglic, R. Lavendomme, *J. Appl. Crystallogr.* 2022, **55**, 1033.
15. P. Mal, D. Schultz, K. Beyeh, K. Rissanen, J. R. Nitschke, *Angew. Chem. Int. Ed.* 2008, **47**, 8297.
16. T. K. Ronson, C. Giri, N. Kodiah Beyeh, A. Minkkinen, F. Topić, J. J. Holstein, K. Rissanen, J. R. Nitschke, *Chem. Eur. J.* 2013, **19**, 3374.
17. A. Riddell, M. M. J. Smulders, J. K. Clegg, J. R. Nitschke, *Chem. Commun.* 2011, **47**, 457.
18. L. Ma, C. J. E. Haynes, A. B. Grommet, A. Walczak, C. C. Parkins, C. M. Doherty, L. Longley, A. Tron, A. R. Stefankiewicz, T. D. Bennett, et al., *Nat. Chem.* 2020, **12**, 270.

CRediT:

Conceptualization (JRN, SM), Investigation (SA, HPR, LM, TKR, BR), Resources (BR, JRN, SM), Visualization (SA, HPR, LM), Writing – Original Draft Preparation (SA), Writing – Review & Editing (SA, HPR, LM, TKR, BR, JRN, SM).
